# Socioeconomic indicators in epidemiologic research: A practical example from the LIFEPATH study

**DOI:** 10.1371/journal.pone.0178071

**Published:** 2017-05-30

**Authors:** Angelo d’Errico, Fulvio Ricceri, Silvia Stringhini, Cristian Carmeli, Mika Kivimaki, Mel Bartley, Cathal McCrory, Murielle Bochud, Peter Vollenweider, Rosario Tumino, Marcel Goldberg, Marie Zins, Henrique Barros, Graham Giles, Gianluca Severi, Giuseppe Costa, Paolo Vineis, LIFEPATH Consortium

**Affiliations:** 1 Epidemiology Unit, ASL TO3, Piedmont Region, Grugliasco, Torino, Italy; 2 Department of Clinical and Biological Science, University of Turin, Turin, Italy; 3 Institute of Social and Preventive Medicine and Department of Psychiatry and Department of Internal Medicine, Lausanne University Hospital, Lausanne, Switzerland; 4 University College London, Department of Epidemiology and Public Health, London, United Kingdom; 5 Clinicum, Faculty of Medicine, University of Helsinki, Helsinki, Finland; 6 The Irish Longitudinal Study on Ageing (TILDA), Trinity College Dublin, Ireland; 7 Cancer Registry, Department of Prevention, ASP, Ragusa, Italy; 8 Population-based Epidemiological Cohorts Unit, INSERM UMS 11, Villejuif, France; 9 Paris Descartes University, Paris, France; 10 EPIUnit- Institute of Public Health, University of Porto, Porto, Portugal; 11 Cancer Epidemiology Centre, Cancer Council Victoria, Melbourne, Australia; 12 Centre de Recherche en Épidémiologie et Santé des Populations (CESP, Inserm U1018), Université Paris-Saclay, UPS, USQ, Gustave Roussy, Villejuif, France; 13 Human Genetics Foundation (HuGeF), Turin, Italy; 14 MRC-PHE Centre for Environment and Health, School of Public Health, Department of Epidemiology and Biostatistics, Imperial College London, London, United Kingdom; National Institute of Health, ITALY

## Abstract

**Background:**

Several social indicators have been used in epidemiological research to describe socioeconomic position (SEP) of people in societies. Among SEP indicators, those more frequently used are education, occupational class and income. Differences in the incidence of several health outcomes have been reported consistently, independently from the indicator employed. Main objectives of the study were to present the socioeconomic classifications of the social indicators which will be employed throughout the LIFEPATH project and to compare social gradients in all-cause mortality observed in the participating adult cohorts using the different SEP indicators.

**Methods:**

Information on the available social indicators (education, own and father’s occupational class, income) from eleven adult cohorts participating in LIFEPATH was collected and harmonized. Mortality by SEP for each indicator was estimated by Poisson regression on each cohort and then evaluated using a meta-analytical approach.

**Results:**

In the meta-analysis, among men mortality was significantly inversely associated with both occupational class and education, but not with father’s occupational class; among women, the increase in mortality in lower social strata was smaller than among men and, except for a slight increase in the lowest education category, no significant differences were found.

**Conclusions:**

Among men, the proposed three-level classifications of occupational class and education were found to predict differences in mortality which is consistent with previous research. Results on women suggest that classifying them through their sole SEP, without considering that of their partners, may imply a misclassification of their social position leading to attenuation of mortality differences.

## Introduction

Socioeconomic position (SEP) is the general term used to refer to the most common forms of inequality. These are usually accepted to be income, wealth, status (or prestige) and social class. These dimensions are correlated in different ways according to the social stratification mechanisms operating in a determined society. There is a longstanding debate on how to measure the rank of individuals in a society, which has its roots in social class theory (see for example: Wright, 2005) [1]. Different measures have been employed in epidemiological research to assess socioeconomic position (SEP). In many studies where measures of income, status and class are not available, educational level is frequently used as the social position indicator [2] and it tends to be correlated empirically with the other measurements [3,4].

Differences in health by occupational class, education and income have been reported consistently for several health outcomes, including self-reported health, chronic and long-term health conditions, and mortality [5–18].

In most theoretical models the occupation performed by people is a central element for attributing them a social position, based on the consideration that in market economies life chances of individuals are mainly determined by their position in the labour market and in the occupational division of labour. Two main sociological schools can be distinguished, one defining SEP in terms of status or prestige attributes [19], the other one through people’s relational power in society [20].

The conceptualization of social position as represented by prestige or social status derives from the functionalist tradition, largely based on Durkheim’s work in Europe [21] and Parsons’ in the U.S. [22], which both consider society as a “living organism”, whose functioning is provided by the different parts. The social stratification of the different occupational groups derives from their functions in society: social roles needing higher skills are more highly remunerated by society in terms of income and social respect [19]. A limitation of the prestige-based measures is that they are represented by continuous scales, such as SIOPS [23], the Cambridge Scale [24], SEI [25], which may lack conceptual clarity on the different social strata, in terms of their number and the cut-offs separating them. In theoretical terms, questions have been raised in relation to the degree of consensus that is assumed by prestige measures of this kind, and the absence of any consideration of power or conflict [26].

**Fig 1:**
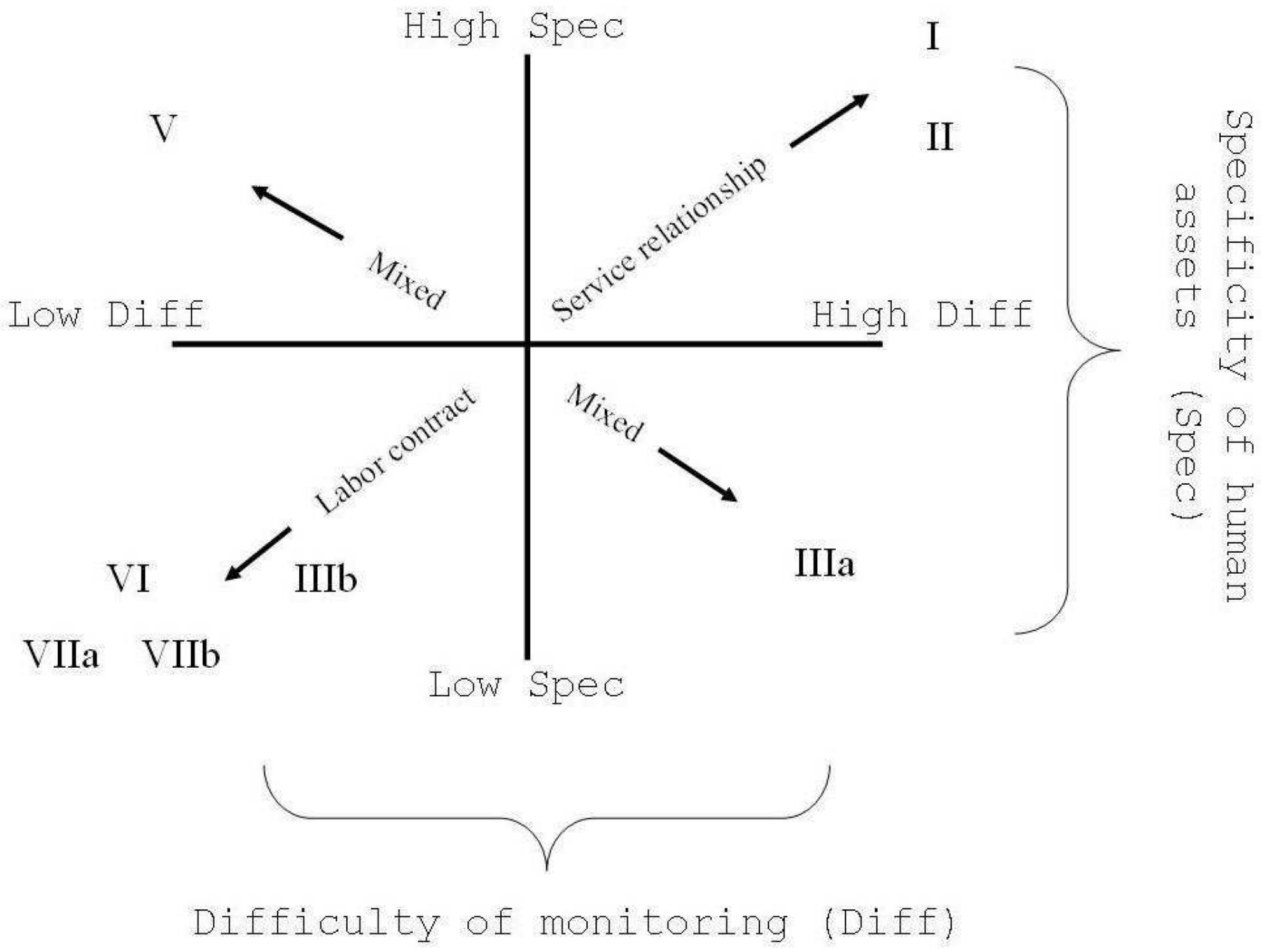
Dimension of work as sources of contractual hazard, forms of employment contract, and location of employee classes of the schema (adapted from Goldthorpe 2000, p. 223, fig 10.2).

The second way of conceptualizing social position derives from Weber’s social theory, in which classes are identified through the level (or probability) of access to economic resources their members have, defined by Weber as “life chances” [20]. Starting from his work, the neo-Weberian school has developed a social classification based on people’s “life chance” in the labour market and at work [27], according to which people are classified both through their educational credentials, which mainly determine their success in the labour market, and their occupation. The Erikson & Goldthorpe (EG) schema, besides distinguishing employers from employees, also keeping separate, within employers, large from small employers and from self-employed workers, categorizes the employees through the nature of their relationship with the employer, discerning those having a “service relationship”, characterized by higher workers’ skills, knowledge, autonomy, salary, benefits, job security and career prospects, from those having a “labour relationship”, characterized by exchange of effort, often physical, with salary, lower wages, higher job insecurity and tighter supervision. The degree of each type of relationship in a certain occupation determines its position on a seven-class ordinal scale, which reaches eleven classes in its most disaggregated form [27]. This is the model that has influenced the way of conceptualizing and measuring socioeconomic position (SEP) in Europe [10,12,28–31], from which derived the European Socio-economic Classification—ESeC, that is our conceptual reference for classifying occupations in the LIFEPATH project. ESeC was built on the same principles of the EG classification (1992) and closely resembles it, classifying occupations in nine ordinal categories based on similarity of resources, in terms of opportunities and ‘life chances’, including employment relations and conditions (Fig 1). By defining what social class is, ESeC also defines what is not, allowing to conceptualize separately the position in the division of labour from that related to education and income.

Education is a stable indicator over time, changing little during adulthood, and in most nations and social groups it is highly correlated with social class, status and income. Education also allows classifying all subjects in a society, independently from their participation in the labour market [32]. Regarding education as a separate phenomenon to socioeconomic position has many advantages. As well as being a strong predictor of occupation and income in adulthood, education also reflects childhood and adolescent SEP, being strongly influenced by material and cultural resources of the family of origin. Therefore, its influence on health could be attributable to many life course processes that are distinct from adult socioeconomic position, such as the long-term effects of early life circumstances. Part of the association between health and education may be due to social selection of those experiencing ill health during childhood into lower educational groups. Indeed, poor health in early life could both limit educational attainment and increase the likelihood of morbidity and premature mortality in adulthood. However, with respect to occupation and income, which can be affected by poor health during adulthood leading to the possibility of reverse causation, educational level has the advantage of being unlikely to be influenced by health conditions occurred in adulthood. A major problem in using educational level as a SEP indicator is that its meaning varies among different birth cohorts, because of changes in educational systems and in the extent of the diffusion of educational credentials in the population.

Income can affect health in two main ways: providing material resources for living, such as those needed to obtain decent housing, clothing and food, or for having access to health care. It also provides opportunities for the household members to avail of professional services (e.g. domestic help, child care, household maintenance) and to participate in social activities, such as cultural events, sports, friends and family gathering, and more in general to exert control over one’s life [33]. Experience of long-term deprivation has been reported as particularly health-damaging, which supports the role of accumulated financial strain across the life course on health [34].

In many studies health status during adulthood has also been associated with early circumstances of social disadvantage (see the reviews by Galobardes et al. [35,36]), mostly measured through father’s or mother’s occupation or educational level. Disadvantaged socioeconomic position in childhood appears to have a stronger influence on the occurrence of certain diseases, such as stroke or stomach cancer, whose determinants would act predominantly during this period of life, whereas for other disorders the relevant exposures would mainly occur later, during adulthood, or exposures in both periods would be important for other disorders, as for coronary heart disease, lung cancer and other respiratory diseases [37–40].

In summary, social gradients in health have been consistently found using occupational class, educational level, income or household financial resources, or parents’ SEP (occupation or education), with some differences related to the health outcome investigated.

Though epidemiology has extensively investigated chemical, physical and behavioural risk factors (smoking, diet, alcohol, physical exercise, occupational factors), in several studies these still explain less than half of the socioeconomic differences in mortality and morbidity [8,14,41], although strong variations are observed by country, depending on the existing social gradient in terms of distribution (prevalence) of chemical, physical and behavioural exposures by social group [42,43]. What is missing in linking SEP with health is an understanding of the intermediate mechanisms and pathways that relate less advantaged SEP with deterioration of organic parameters. For example, research on immune markers in the Whitehall II study has shown that glucocorticoids and inflammation may in part explain how the body mediates the effects of social and economic disadvantage thus leading to disease, and this is partly independent from common/known risk factors [44,45]. More generally, recent studies have shown that SEP can influence the global physiological dysregulation across the life-course, measured using allostatic load, a measure of biological multisystem wastage [46–48].

The study of the biological mechanisms through which SEP influences health, with a particular focus on healthy ageing, is the main aim of the EU-funded LIFEPATH project, of which the present study is part.

Aims of this paper are: 1) to present the socioeconomic classifications of occupational class, education and income which will be employed throughout the EC Horizon 2020 LIFEPATH project to categorize the socioeconomic position of the population enrolled, as well as the distribution of these SEP indicators in the different cohorts, and 2) to compare social gradients in all-cause mortality observed in the adult cohorts using the different SEP indicators, both in the individual cohorts and overall, through a meta-analytic process.

## Methods

### Cohorts

LIFEPATH is a scientific project funded by the European Community that is devoted to the investigation of the biological pathways underlying social differences in healthy aging. The specific objectives of this project, that integrates social science approaches with biology (including molecular epidemiology) using existing population cohorts and omics measurements (particularly epigenomics), are to show that healthy ageing is an achievable goal for society, as it is already experienced by individuals of more advantaged SEP, and to improve the understanding of the mechanisms through which healthy ageing pathways diverge by SEP, by investigating life course biological pathways using omic technologies.

**Fig 2:**
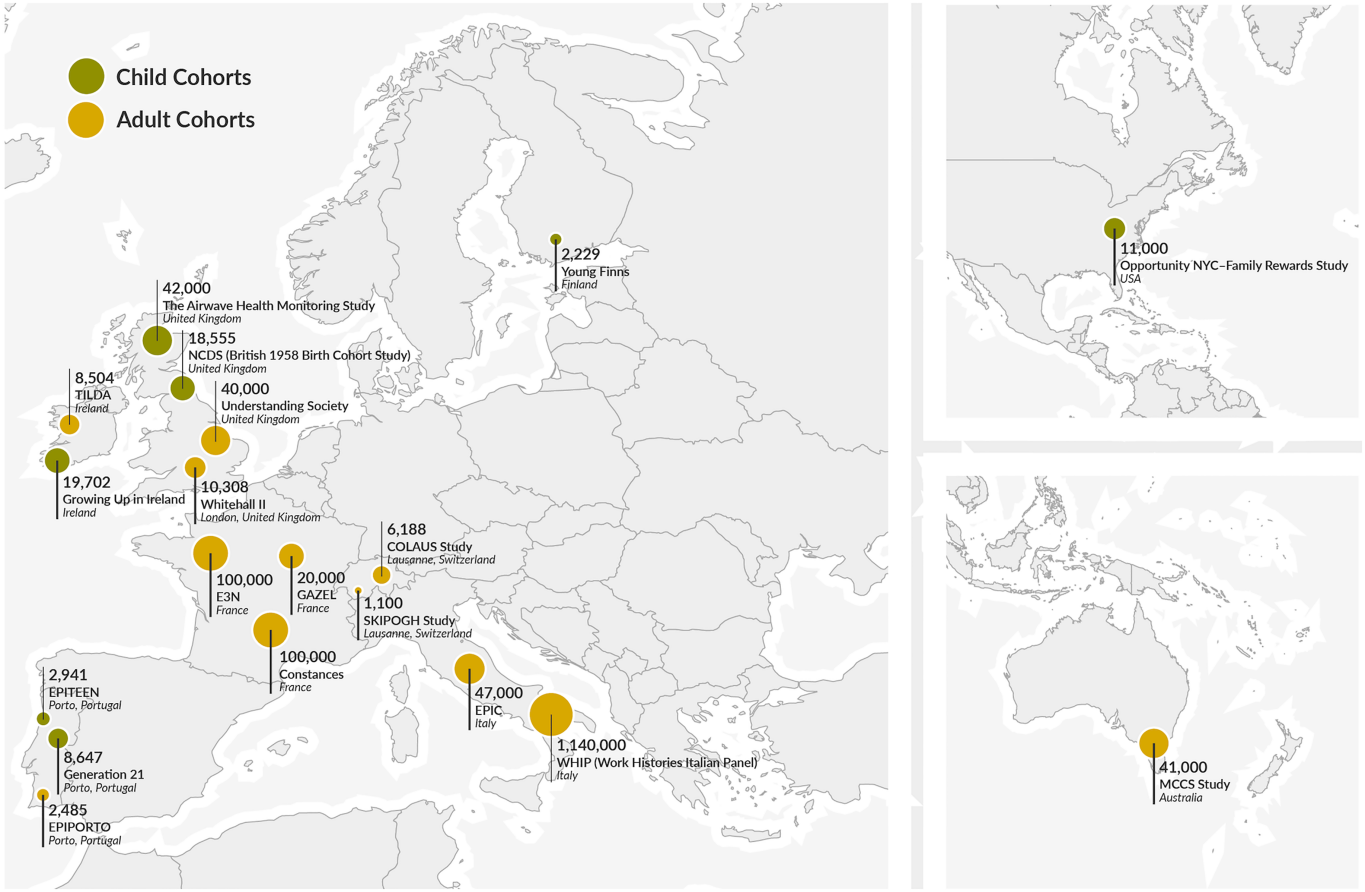
Lifepath distribution of cohorts.

For this purpose, a consortium of cohorts was built, including seven child cohorts and eleven adult cohorts (Fig 2). To merge and analyze together data from the different LIFEPATH cohorts, information on occupational class, education, father’s occupational class and income had to be harmonized. The harmonization was performed in the initial phases of the project, in order to avoid post hoc decision making on SEP measures categorization in the different cohorts, which may give authors the possibility of reformulating their exposure definition during the analytic process [49].

Given that SEP indicators relevant for adults differ from those important to characterize child SEP, in this paper we will focus only on the adult cohorts, whose detailed description is available in Table 1 and more details can be found in the S1 File. Briefly, the consortium is composed of one Portuguese cohort (Epiporto [50], participants randomly selected within Porto dwellers), three French cohorts (Constances [51], adult subjects randomly selected from French adults; E3N [52], volunteers from the French National school system; Gazel [53], workers of the French national gas and electricity company), two Italian cohorts (EPIC-Italy [54], volunteers from 4 Italian cities; WHIP-retired [55], random sample of workers retired from private enterprises), two Swiss cohorts (CoLaus/PsycoLaus [56], random sample of Lausanne inhabitants; Skipogh [57], Swiss volunteer families), one Irish cohort (TILDA [58], random sample of community dwelling older persons aged 50 years+), one English cohort (Whitehall II [59], London-based civil servants), and one Australian cohort (MCCS [60], random sample of Melbourne dwellers), for a total of 518,061 participants.

**Table 1:** Description of the adults’ cohorts included in LIFEPATH projects.

Name	Country	Types of subjects	Recruitment	N of subjects (N of death)	Mean age (SD)	% males	Median years FU /person years	References
CoLaus	Switzerland	Random sample of Lausanne inhabitants	2003–2006	6,733 (210)	52.6 (10.7)	47.4	6.45 / 40,990	Firmann et al, 2008 [56]
Constances	France	Random sample of French adults	2012-ongoing	71,500 (NA)	48.2 (13.8)	45.9	Ongoing	Zins et al, 2015 [51]
E3N	France	Volunteers (National school system)	1989–1991	98,995 (9,075)	49.4 (6.7)	0.00	17.99 / 1,604,456	Clavel-Chapelon et al., 1997 [52]
EPIC-Italy	Italy	Volunteers (from Turin, Varese, Naples, and Ragusa)	1993–1998	34,151 (2,000)	49.6 (8.0)	34.1	15.88 / 533,429	Palli et al, 2003 [54]
EPIPORTO	Portugal	Random sample of Porto inhabitants	1995–2005	2,485 (236)	52.9 (15.5)	38.1	6.11 / 11,991	Santos et al, 2012 [50]
Gazel	France	Workers of the French national gas and electricity company	1989	20,625 (2,456)	43.7 (3.5)	72.8	26.74 / 525,085	Goldberg et al, 2007 [53]
MCCS	Australia	Volunteers (from Melbourne city)	1990–1994	41,514 (9,122)	55.4 (8.7)	41.1	18.00 / 724,866	Hodge et al., 2013 [60]
Skipogh	Switzerland	Volunteer families	2009–2013	1,153 (9)	47.4 (17.5)	47.5	2.82 / 2,305	Alwan et al., 2014 [57]
Tilda	Ireland	Volunteers >50 years	2009–2011	8,504 (NA)	63.0 (9.4)	44.5	NA	Whelan & Savva, 2013 [58]
WHIP-retired	Italy	Random sample of retired not-public employed	1990–2012	223,586 (32,139)	57.8 (3.8)	65.4	11.59 / 2,641,599	Filippi et al., 2012 [55]
Whitehall II	United Kingdom	London-based civil servant	1991–1994	8,815 (1,149)	50.3 (6.1)	68.7	20.39 / 179,042	Marmot et al, 1991 [59]

Each cohort was approved by the appropriate Ethical Committee. Details are provided in S1 File, together with the cohorts’ description. All investigations have been conducted according to the principles expressed in the Declaration of Helsinki. Written informed consent has been obtained from the participants.

### Variables harmonization

#### Educational level

Educational level is available in all LIFEPATH cohorts, except for WHIP-retired. Educational systems are nation-specific and each cohort collected data in different ways (most of the cohorts collected information about the level of education achieved with different depth, and Epiporto about years of school only). Despite that, it was possible to identify at least three levels that were comparable across countries: 1) primary and lower secondary school (from 7 to 9 years after the kindergarten with a basic curriculum in languages, mathematics and other subjects), 2) higher secondary school (around 4–5 years more, high school diploma level) and 3) tertiary education (any degree after high school, such as BSc, MSc, and further education). Primary education was not kept separate from low secondary, because information on the former was available only for few cohorts (E3N, EPIC-Italy and Gazel). In some countries (Italy, France, and Switzerland), it was also possible to identify a subgroup of participants that attended a vocational school for professional training (2–3 years after lower secondary school).

#### Occupation

Occupation was collected in all cohorts, except for the Australian cohort (MCCS), where information was collected on employment status and not about type of occupation. A simple dichotomous variable regarding the employment status was created. For a few cohorts (MCCS, Skipogh, TILDA, Constances) there was also information on different types of non-employment (retired, housewife, disabled, unemployed).

We decided to harmonize participants’ current occupation and participants’ last-known occupation (before retirement, before unemployment, etc.) using two different variables. First, a dichotomous variable distinguishing between manual and non-manual workers was created. Then, all occupations were classified using the ESeC (Table 2). Because information about occupation within LIFEPATH cohorts was collected with different depth, it was not possible to use the 9-categories detail of ESEC classification and, according to the available data, we decided to group the ESeC classes into three categories:

- Higher occupations including:
  - E-SeC class 1: large employers, higher professionals and managers;
  - E-SeC class 2: lower professionals and managers, and higher grade technical and supervisory occupations;
  - E-SeC class 3: higher grade clerical, services and sales workers;
- Intermediate occupations including:
  - ESeC class 4: small employers and self-employed outside of agriculture;
  - E-SeC class 5: farmers, self employed in agriculture;
  - E-SeC class 6: lower supervisory and technical occupations;
- Routine and manual occupations including:
  - ESeC class 7: lower clerical, services, and sales workers;
  - E-SeC class 8: skilled workers;
  - E-SeC class 9: semi- and unskilled workers.

**Table 2:** European Socio-economic Classification.

ESeC Class		Common Term	Employment regulation	3- class schema
1	Large employers, higher grade professional, administrative and managerial occupations	Large employers and higher salariat	Owners and service relationship	Large employers and salariat
2	Lower grade professional, administrative and managerial occupations and higher grade technician and supervisory occupations	Lower salariat	Service relationship	
3	Intermediate occupations	Higher grade white collar workers	Mixed	
4	Small employer and self employed occupations (excluding agriculture etc)	Petit bourgeoisie or independents	Owners	Intermediate
5	Self employed occupations (agriculture etc)	Petit bourgeoisie or independents	Owners	
6	Lower supervisory and lower technician occupations	Higher grade blue collar workers	Mixed	
7	Lower services, sales and clerical occupations	Lower grade white collar workers	Labour contract	Routine and manual
8	Lower technical occupations	Skilled workers	Labour contract	
9	Routine occupations	Semi- and non-skilled workers	Labour contract	

The three-category variable was judged the most detailed possibility of reclassifying participants’ occupations, in order to avoid exclusion of cohorts or excessive misclassification among classes.

#### Father’s occupation

In order to infer information about socioeconomic position in childhood, some cohorts collected also information on the main occupation of participants’ fathers. Two cohorts did not collect this information (CoLaus/PsycoLaus and WHIP-retired) and other two used categories of father’s occupational class less detailed than those used to collect participants’ occupation (Gazel and Skipogh), although sufficiently enough to classify subjects according to the same two- and three- occupational classes used for own occupation.

#### Income

Only in three cohorts information about income was collected and, furthermore, it was done differently: in the TILDA cohort it was collected asking participants their precise yearly income, in CoLaus/PsycoLaus it was collected using categories, while in the WHIP-retired cohort the exact income amount was available from administrative records. Since mean amount of salary was different across countries and time period, we decided to harmonize it using cohort-based quintiles.

Harmonization was performed using SAS 9.2 software and all harmonized databases are stored at the University of Turin.

### Mortality assessment

Each cohort provided participants’ vital status, follow-up time, and eventual mortality date. In most cohorts vital status was assessed through record linkage with administrative data. In CoLaus/PsycoLaus it was assessed through active follow-up.

### Statistical analysis

Data were described using absolute frequencies and percentages or means and standard deviations for categorical or continuous variables, respectively. Correlations among SEP variables were tested using the Spearman co-graduation coefficient, due to the ordinal behaviour of the variables.

For each harmonized SEP variable, the impact on mortality was tested using a Poisson model with the Huber estimator of the variance [61], adjusted for age, separately for gender and cohort. Constances, Skipogh and TILDA cohorts were not included in the analyses on mortality, the former two because follow-up duration was too short to observe a sufficient number of deaths, while for TILDA mortality had not been made available to the LIFEPATH project. Results coming from the different cohorts were pooled through a meta-analytic process with random effects, using the DerSimonian & Laird weights [62]. Heterogeneity among cohorts was tested using the Higging heterogeneity index (I^2^) and the Cochrane’s *Q* test based upon inverse variance weights.

A 5% level of significance was considered for all tests. All analyses were performed using STATA v.13.

## Results

### Harmonization

Harmonization of the available variables was done for each cohort and a detailed codebook can be found in the S2, S3 and S4 Files, for education level, subjects’ occupation, and fathers’ occupation respectively. In Tables 3 (males) and 4 (females), numbers of participants in each socioeconomic category are presented separately for each cohort. Results across genders were similar, although women showed in general slightly higher proportions in lower SEP categories.

**Table 3:** Baseline distribution of subjects and deaths in each socioeconomic category stratified by cohort (Males).

	Colaus			Constances		EPIC Italy			EPIPORTO			GAZEL			MCCS			Skipogh		Tilda		WHIP-retired			Whitehall II		
	N	%	Deaths	N	%	N	%	Deaths	N	%	Deaths	N	%	Deaths	N	%	Deaths	N	%	N	%	N	%	Deaths	N	%	Deaths
**Education**																											
**Three levels variable**																											
*primary or lower secondary school*	1,704	53.4	88	8,877	31.6	6,400	57.0	599	553	58.5	114	10,735	73.0	1582	10,179	59.7	3397	246	46.4	2,109	55.8				1,755	30.5	251
*higher secondary school*	448	14.1	17	4,472	15.9	3,232	28.8	192	148	15.6	212	1,265	8.6	167	1,863	10.9	532	163	30.8	1,066	28.2				1,688	29.4	199
*tertiary education*	1,030	32.3	31	14,716	52.4	1,597	14.2	75	245	25.9	12	2,705	18.4	257	4,998	29.3	910	121	22.8	604	16.0				2,304	40.1	259
*missing values*	7	0.2																									
**Four levels variable**																											
*primary or lower secondary school*	550	17.3	28	2,881	10.3	4,614	41.1	462				2,920	19.9	489				56	10.6								
*volational school*	1,154	36.2	60	5,996	21.4	1,786	15.9	137				7,815	53.2	1093				190	35.9								
*higher secondary school*	448	14.1	17	4,472	15.9	3,232	28.8	192				1,265	8.6	167				163	30.8								
*tertiary education*	1,030	32.3	31	14,716	52.4	1,597	14.2	75				2,705	18.4	257				121	22.8								
*missing values*	7	0.2																									
**Employment status**																											
**Two levels variable**																											
*Employed*	2,438	76.6	49	18,958	64.4	8,534	73.4	457	551	58.4	36				6,931	49.7	687	378	70.7	1,579	42.2				5,733	94.7	707
*Not employed*	747	23.4	87	10,463	35.6	3,093	26.6	453	393	41.6	109				7,022	50.3	2935	157	29.4	2,165	57.8				324	5.4	68
*missing values*	4	0.1																									
**Five levels variable**																											
*Employed*				18,958	66.3										6,931	49.8	687	378	75.0	1,579	42.3						
*Not employed*: *retired*				7,214	25.2										6,249	44.9	2086	94	18.7	1,683	45.1						
*Not employed*: *housewife*				295	1.0										103	0.7	20	11	2.2	32	0.9						
*Not employed*: *unemployed*				1,823	6.4										638	4.6	108	20	4.0	258	6.9						
*Not employed*: *disabled*				305	1.1													1	0.2	181	4.9						
*missing values*																											
**Current job**																											
**Two levels variable**																											
*Manual workers*	621	19	13	2,714	15.2	3,231	37.9	186	148	26.9	10	2,016	14.1	402				70	18.8	856	67.4				381	6.7	74
*Non manual workers*	1,814	57	35	15,195	84.9	5,303	62.1	271	403	73.1	26	12,274	85.9	1562				302	81.2	415	32.7				5,352	93.4	633
*missing values*	754	24																									
**Three levels variable**																											
*Classes 1–3 ESEC*	575	18	8	7,665	42.8	863	10.1	38	144	26.1	5	4,060	28.4	414				113	30.4	339	26.7				3,735	65.2	421
*Classes 4–6 ESEC*	809	25	15	4,826	27.0	4,525	53.0	540	163	29.6	9	7,351	51.4	1021				88	23.7	489	38.5				1,617	28.2	212
*Classes 7–9 ESEC*	1,051	33	26	5,418	30.3	3,146	36.9	179	244	44.3	22	2,879	20.2	529				171	46.0	443	34.9				381	6.7	74
*missing values*	754	24																									
**Current/last job**																											
**Two levels variable**																											
*Manual workers*	548	17.2	14	4,643	17.0	4,851	42.0	432	299	32.8	53	2,016	14.1	402				103	20.8	1,623	58.1	85,499	66.6	15298	416	6.9	90
*Non manual workers*	1,973	61.9	42	22,618	82.9	6,714	58.1	462	613	67.2	91	12,274	85.9	1562				393	79.2	1,171	41.9	42,900	33.4	5752	5,641	93.1	685
*missing values*	668	21.0																									
**Three levels variable**																											
*Classes 1–3 ESEC*	592	18.6	9	10,871	39.9	1,025	8.9	63	189	20.7	15	4,060	28.4	414				143	28.8	903	32.3	7,655	6.0	561	3,918	64.7	451
*Classes 4–6 ESEC*	828	25.9	16	7,713	28.3	5,799	50.1	412	256	28.1	41	7,351	51.4	1021				118	23.8	560	20.0	35,245	27.5	5191	1,726	28.5	234
*Classes 7–9 ESEC*	1,101	34.5	30	8,677	31.8	4,741	41.0	419	467	51.2	88	2,879	20.2	529				235	47.4	1,331	47.6	85,499	66.6	15298	416	6.9	90
*missing values*	668	21.0																									
**Father's job**																											
**Two levels variable**																											
*Manual workers*	NA			9,992	39.1	8,146	71.3	638	310	50.9	34	8,730	65.2	1159				124	36.5	1,864	71.3				1,759	41.7	252
*Non manual workers*				15,542	60.9	3,277	28.7	252	299	49.1	28	4,657	34.8	635				216	63.5	749	28.7				2,456	58.3	289
*missing values*																											
**Three levels variable**																											
*Classes 1–3 ESEC*	NA			5,214	20.4	1,459	5.1	36	91	14.9	4	4,657	34.8	635				72	21.2	444	27.7				409	9.7	49
*Classes 4–6 ESEC*				10,152	39.8	12,480	43.7	411	126	20.7	13	2,985	22.3	371				130	38.2	130	21.5				1,337	31.7	142
*Classes 7–9 ESEC*				10,168	39.8	14,634	51.2	443	392	64.4	45	5,745	42.9	788				138	40.6	2,039	50.8				2,469	58.6	350
*missing values*																											
**Income**																											
**Quintiles of income**																											
*1st quintile*	305	17.7	12																	477	16.5	8,721	6.0	2071			
*2nd quintile*	349	20.3	11																	326	11.3	26,226	18.0	6062			
*3rd quintile*	316	18.4	3																	622	21.5	33,399	22.9	6251			
*4th quintile*	268	15.6	6																	744	25.7	38,058	26.0	5933			
*5th quintile*	483	28.1	5																	729	25.2	39,878	27.3	5818			
*missing values*																											

**Table 4:** Baseline distribution of subjects and deaths in each socioeconomic category stratified by cohort (Females).

	Colaus			Constances		E3N			EPIC Italy			EPIPORTO			GAZEL			MCCS			Skipogh		Tilda		WHIP-retired			Whitehall II		
	N	%	Deaths	N	%	N	%	Deaths	N	%	Deaths	N	%	Deaths	N	%	Deaths	N	%	Deaths	N	%	N	%	N	%	Deaths	N	%	Deaths
**Education**																														
**Three levels variable**																														
*primary or lower secondary school*	2,070	58.5	53	8,267	25.1	4,909	5.2	702	14,567	65.9	765	963	62.6	78	4,172	76.5	299	16,770	68.6	3284	288	50.0	2,383	50.5				1,385	54.8	199
*higher secondary school*	440	12.4	9	6,195	18.8	55,649	58.7	5182	4,992	22.6	198	172	11.2	5	661	12.1	42	2,260	9.2	385	187	32.5	1,729	36.6				507	20.1	66
*tertiary education*	1,027	29.0	11	18,475	56.1	34,176	36.1	2735	2,546	11.5	94	404	26.3	6	621	11.4	35	5,435	22.2	611	101	17.5	609	12.9				634	25.1	57
*missing values*																														
**Four levels variable**																														
*primary or lower secondary school*	847	24.0	29	3,567	10.8	4,909	5.2	702	12,232	55.3	675				1,508	27.7	113				78	13.5								
*volational school*	1,223	34.6	24	4,700	14.3	8,435	8.9	874	2,335	10.6	90				2,664	48.8	186				210	36.5								
*higher secondary school*	440	12.4	9	6,195	18.8	47,214	49.8	4308	4,992	22.6	198				661	12.1	42				187	32.5								
*tertiary education*	1,027	29.0	11	18,475	56.1	34,176	36.1	2735	2,546	11.5	94				621	11.4	35				101	17.5								
*missing values*																														
**Employment status**																														
**Two levels variable**																														
*Employed*	2,117	59.8	18	21,839	63.3	73,287	77.1	4953	7,645	43.9	231	755	49.1	7				7,489	36.9	444	372	63.7	1,562	33.6				2,579	93.5	329
*Not employed*	1,424	40.2	55	12,671	36.7	21,756	22.9	3676	9,761	56.1	606	784	50.9	82				12,801	63.1	2790	212	36.3	3,092	66.4				179	6.5	45
*missing values*																														
**Five levels variable**																														
*Employed*				21,839	66.3													7,489	37.1	444	372	67.5	1,562	33.9						
*Not employed*: *retired*				7,433	22.6													6,626	32.8	1732	98	17.8	1,365	29.6						
*Not employed*: *housewife*				1,100	3.3													5,679	28.1	1007	63	11.4	1,314	28.5						
*Not employed*: *unemployed*				2,176	6.6													388	1.9	42	13	2.4	155	3.4						
*Not employed*: *disabled*				370	1.1																5	0.9	214	4.6						
*missing values*																														
**Current job**																														
**Two levels variable**																														
*Manual workers*	957	51.7	9	622	3.0	379	0.6	68	2,583	33.8	91	234	31.0	2	1,499	28.8	100				17	4.7	572	38.8				1,016	39.4	157
*Non manual workers*	893	48.3	6	19,937	97.0	61,734	99.4	6882	5,062	66.2	140	521	69.0	5	3,713	71.2	253				345	95.3	903	61.2				1,563	60.6	172
*missing values*																														
**Three levels variable**																														
*Classes 1–3 ESEC*	202	9.6	1	5,645	27.5	6,640	10.7	1048	373	4.9	12	215	28.5	2	421	8.1	29				60	16.6	460	31.2				606	26.5	51
*Classes 4–6 ESEC*	639	30.3	5	6,880	33.5	44,549	71.7	4583	4,770	62.4	134	126	16.7	1	3,104	59.6	212				151	41.7	218	14.8				957	37.1	121
*Classes 7–9 ESEC*	1,269	60.1	12	8,034	39.1	10,924	17.6	1319	2,502	32.7	85	414	54.8	4	1,687	32.4	113				151	41.7	797	54.0				1,016	39.4	157
*missing values*																														
**Current/last job**																														
**Two levels variable**																														
*Manual workers*	1,217	48.5	9	1,299	4.1				4,707	40.7	296	560	42.4	51	1,499	28.8	100				32	3.3	1,018	37.3	22,283	56.5	1474	1,122	40.7	183
*Non manual workers*	1,293	51.5	13	30,106	95.9				6,856	59.3	275	761	57.6	18	3,713	71.2	253				480	93.8	1,708	62.7	17,154	43.5	956	1,636	59.3	191
*missing values*																														
**Three levels variable**																														
*Classes 1–3 ESEC*	211	9.4	1	7,709	24.6				454	3.9	19	241	18.2	2	421	8.1	29				66	12.9	965	35.4	946	2.4	32	631	22.9	56
*Classes 4–6 ESEC*	672	29.9	7	10,632	33.9				6,527	56.5	266	204	15.4	4	3,104	59.6	212				201	39.3	272	10.0	16,208	41.1	924	1,005	36.4	135
*Classes 7–9 ESEC*	1,367	60.8	14	13,064	41.6				4,582	39.6	286	876	66.3	63	1,687	32.4	113				245	47.9	1,489	54.6	22,282	56.5	1474	1,122	40.7	183
*missing values*																														
**Father's job**																														
**Two levels variable**																														
*Manual workers*				10,966	36.9	28,521	40.0	2369	11,921	69.5	575	499	49.7	13	2,897	59.2	199				145	38.4	2,287	70.3				913	50.7	131
*Non manual workers*				18,725	63.1	42,736	60.0	3649	5,229	30.5	247	506	50.4	13	1,998	40.8	140				233	61.6	967	29.7				889	49.3	108
*missing values*																														
**Three levels variable**																														
*Classes 1–3 ESEC*				6,401	21.6	11,495	16.1	982	938	5.5	43	145	14.4	1	1,998	40.8	140				88	23.3	624	19.2				170	9.4	20
*Classes 4–6 ESEC*				12,188	41.1	30,533	42.9	2534	7,416	43.2	376	216	21.5	6	992	20.3	54				132	34.9	214	6.6				498	27.6	65
*Classes 7–9 ESEC*				11,102	37.4	29,229	41.0	2502	8,796	51.3	403	644	64.1	19	1,905	38.9	145				158	41.8	2,416	74.3				1,134	62.9	154
*missing values*																														
**Income**																														
**Quintiles of income**																														
*1st quintile*	498	26.1	7																				684	21.6	27,971	36.2	2056			
*2nd quintile*	491	25.8	6																				405	12.8	26,487	34.3	2444			
*3rd quintile*	379	19.9	3																				686	21.6	11,277	14.6	792			
*4th quintile*	240	12.6	2																				765	24.1	6,680	8.6	408			
*5th quintile*	299	15.7	0																				633	20.0	4,889	6.3	304			
*missing values*																														

Percentages of participants in the different educational categories, not considering those with missing information, were similar across cohorts (around 50–70% in the lowest category and the remaining more or less equally distributed in the other two categories). There were two exceptions: E3N cohort, with a very low percentage of participants in the lowest category (around 5%) and Constances, with a high percentage of participants in the highest one (more than 50%). The proportion of subjects in the lowest educational category was also quite low in Whitehall II, but only among males (30%), whereas among females it was similar to the other cohorts (55%).

Employment status varied from 100% employed in Gazel (participants were recruited at the Electricity and Gas Company in France) to 0% employed in WHIP-retired, which includes only retired people. Whitehall II study was also an occupational cohort, but we set the baseline at the third wave for reasons of availability of data, and only 95% of participants were still working at that time. In the remaining cohorts, the proportion of participants not in employment varied between 30% to 60%, dependently on the different baseline mean age of the subjects. For the few cohorts for which information on the reason for non-employment was available (Constances, Skipogh, TILDA, MCCS), non-employed were mainly retired people, followed by housewives, while only a small proportion was unemployed or disabled.

Regarding current/last occupation, the proportion of non-manual workers, not considering those with missing information, was above 50% in almost all cohorts for both genders, except WHIP-retired (33% among males and 44% among females) and TILDA (42% among males), with high proportions (above 80%) in Constances, E3N, Skipogh, Gazel and Whitehall II (males only). These cohorts, except for Skipogh, were also the ones showing a low percentage of participants in the lowest occupational class (mainly below 30%), while in all other cohorts it was around 50%, with the highest percentages (around 60%) in Epiporto and WHIP-retired.

In all cohorts for which information on fathers’ occupation was available, the occupations of the participants fell on average in higher occupational classes compared to those of their fathers, with the exception of Skipogh, although with a high variability among cohorts; the proportion of subjects having tertiary education was also increased in most cohorts, although less consistently.

All correlations among socioeconomic indicators in each cohort were statistically significant and were in general quite similar across genders, so they are presented without gender stratification (Table 5). A strong positive correlation between educational level and occupational class was present for almost all cohorts (range of Spearman coefficients: 0.29–0.79), as well as a fair positive correlation between participants’ education and fathers’ occupational class (range of Spearman coefficients: 0.20–0.39), and a mild positive correlation between subjects’ and fathers’ occupational class (range of Spearman coefficients: 0.14–0.36), with the exception of E3N (Spearman coefficients: -0.08 and 0–02, respectively). In the few cohorts where it was available, personal income was fairly positively correlated both with subjects’ education and occupational class.

**Table 5:** Spearman cograduation coefficient measuring correlation among socioeconomic variables in each cohort.

Cohort	Correlation	*Education*^¶^	*Current/last job*^†^	*Father's job*^‡^	Income*
**CoLaus**	*Education*^¶^	1	0.52	—	0.34
	*Current/last job*^†^	0.52	1	—	0.34
	*Father's job*^‡^	—	—	—	—
**Constances**	*Education*^¶^	1	0.56	0.32	—
	*Current/last job*^†^	0.56	1	0.32	—
	*Father's job*^‡^	0.32	0.32	1	—
**E3N**	*Education*^¶^	1	0.08	0.21	—
	*Current/last job*^†^	0.08	1	0.02	—
	*Father's job*^‡^	0.21	0.02	1	—
**EPIC Italy**	*Education*^¶^	1	0.50	0.27	—
	*Current/last job*^†^	0.50	1	0.23	—
	*Father's job*^‡^	0.27	0.23	1	—
**EpiPorto**	*Education*^¶^	1	0.79	0.39	—
	*Current/last job*^†^	0.79	1	0.36	—
	*Father's job*^‡^	0.39	0.36	1	—
**GazEl**	*Education*^¶^	1	0.29	0.20	—
	*Current/last job*^†^	0.29	1	0.14	—
	*Father's job*^‡^	0.20	0.14	1	—
**Skipogh**	*Education*^¶^	1	0.49	0.31	—
	*Current/last job*^†^	0.49	1	0.25	—
	*Father's job*^‡^	0.31	0.25	1	—
**Tilda**	*Education*^¶^	1	0.48	0.31	0.39
	*Current/last job*^†^	0.48	1	0.22	0.25
	*Father's job*^‡^	0.31	0.22	1	0.13
**WHIP-retired**	*Education*^¶^	—	—	—	—
	*Current/last job*^†^	—	1	—	0.53
	*Father's job*^‡^	—	—	—	—
**Whitehall II**	*Education*^¶^	1	0.44	0.27	—
	*Current/last job*^†^	0.44	1	0.27	—
	*Father's job*^‡^	0.27	0.27	1	—

Variable codes:

^¶^Education: 1 = primary or lower secondary school, 2 = higher secondary school, 3 = tertiary education;

^†^Current/last job: 1 = Class 7–9 ESEC (low), 2 = Class 4–6 ESEC (medium), 3 = Class 1–3 ESEC (high);

^‡^Father’s job: 1 = Class 7–9 ESEC (low), 2 = Class 4–6 ESEC (medium), 3 = Class 1–3 ESEC (high);

*Income: 1 = 1st quintile of income (lowest), 2 = 2nd quintile; 3 = 3rd quintile; 4 = 4th quintile; 5 = 5th quintile (highest).

### Mortality

**Fig 3:**
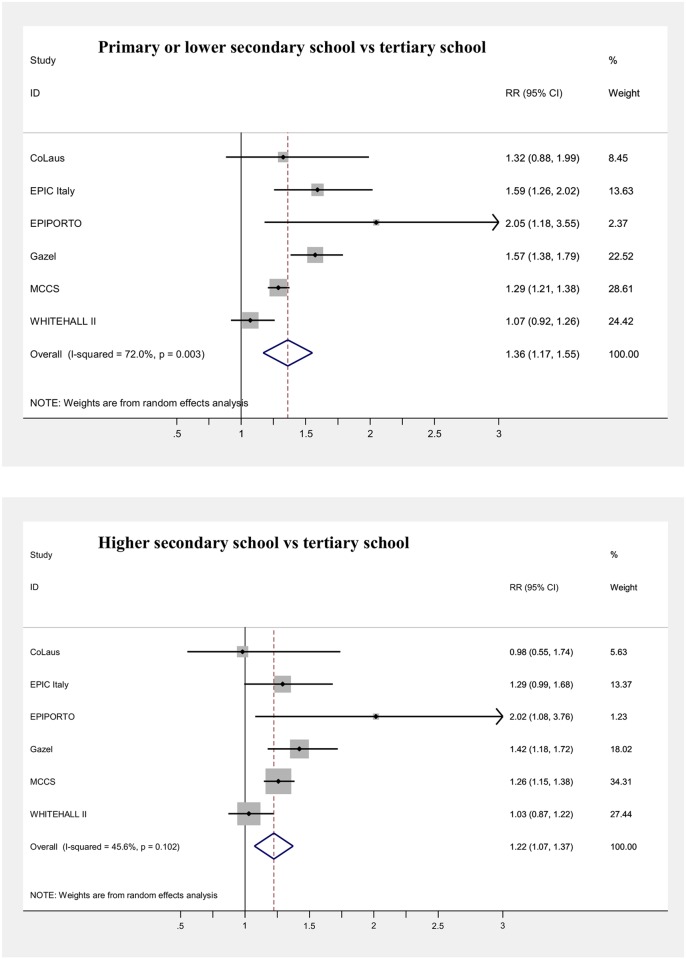
Meta-analysis of the association between education level and mortality (Males).

**Fig 4:**
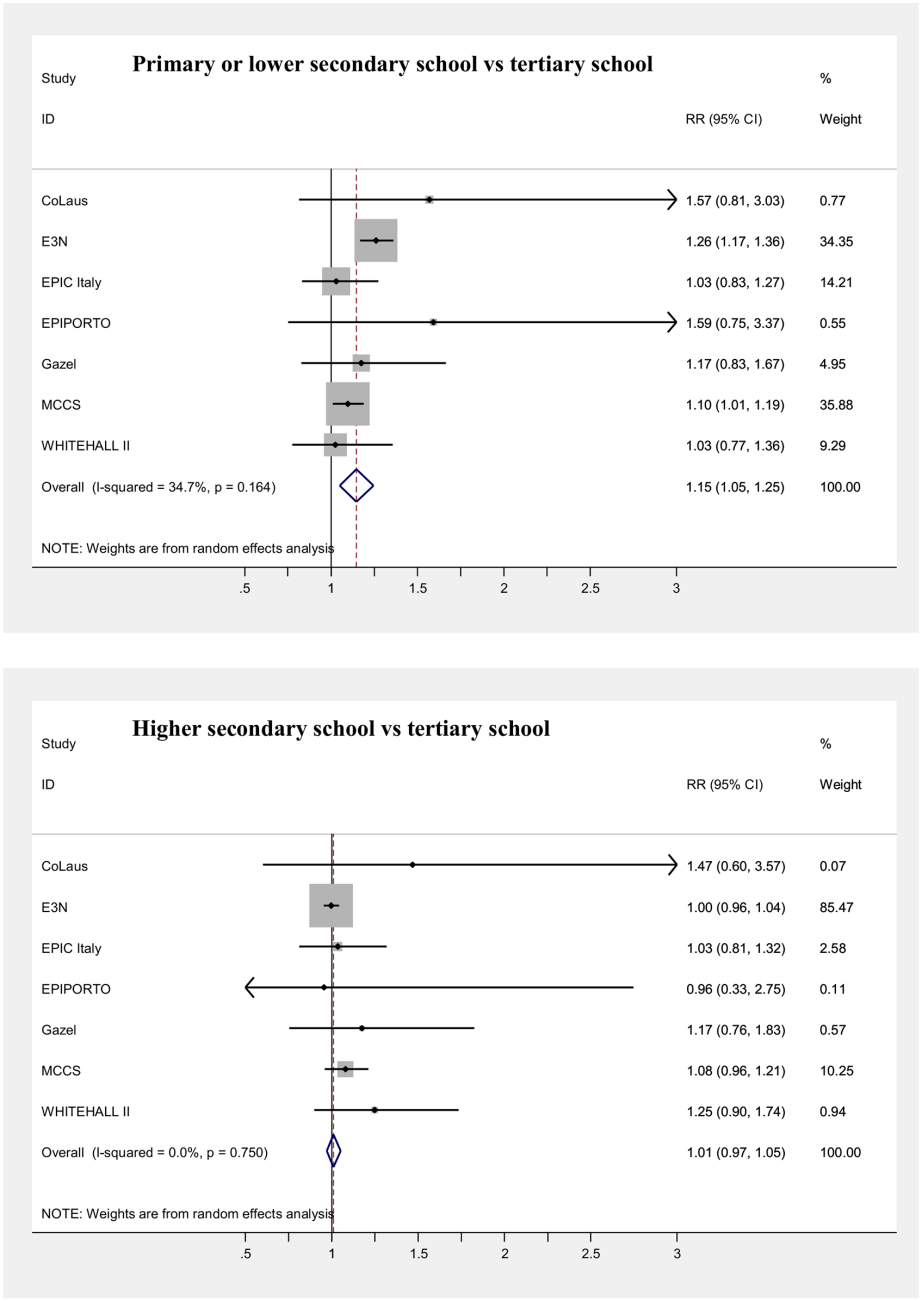
Meta-analysis of the association between education level and mortality (Females).

**Fig 5:**
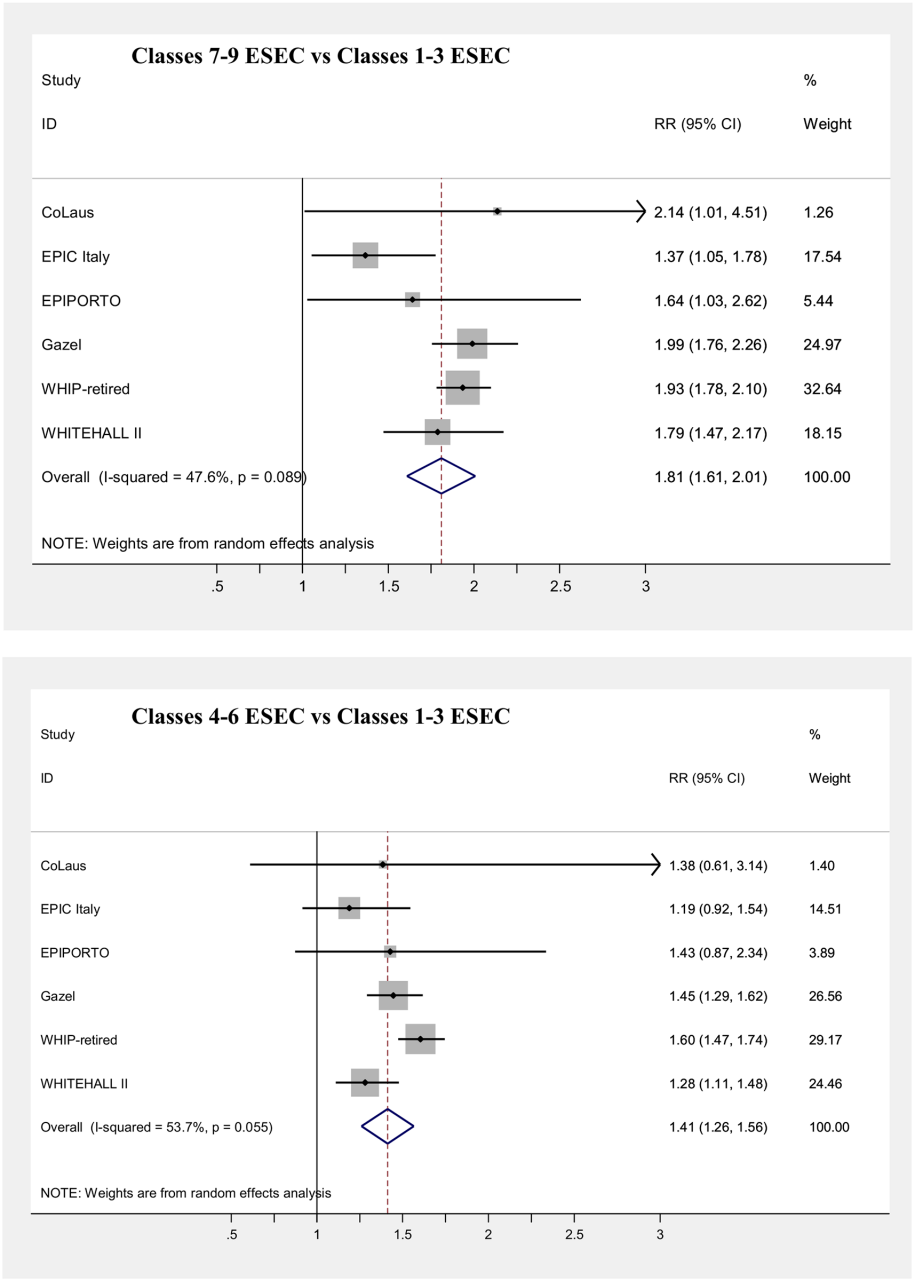
Meta-analysis of the association between current/last job and mortality (Males).

**Fig 6:**
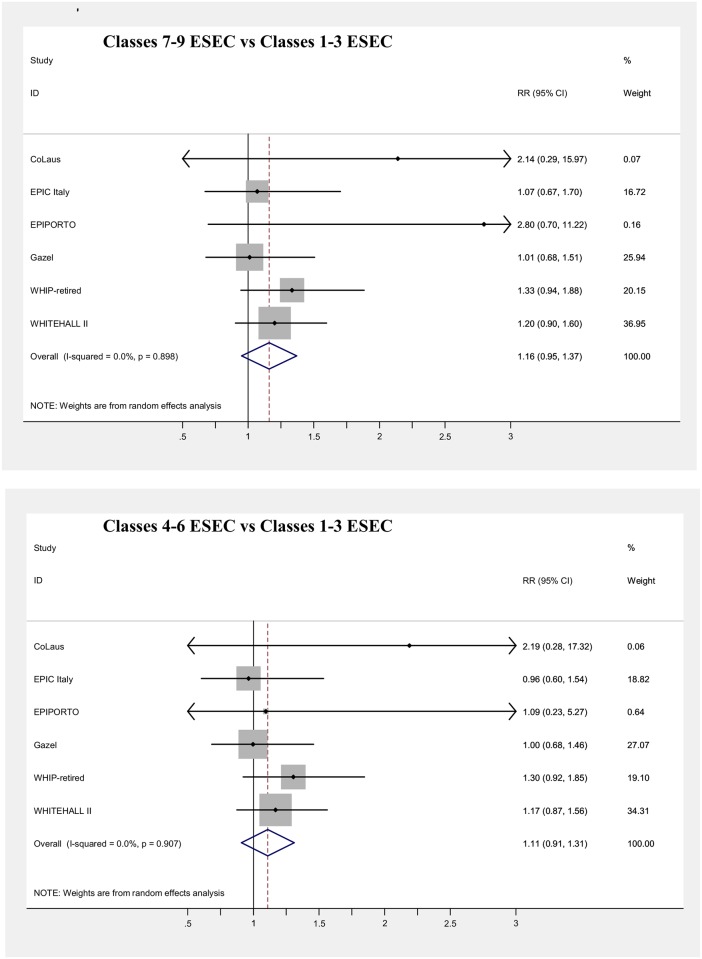
Meta-analysis of the association between current/last job and mortality (Females).

**Fig 7:**
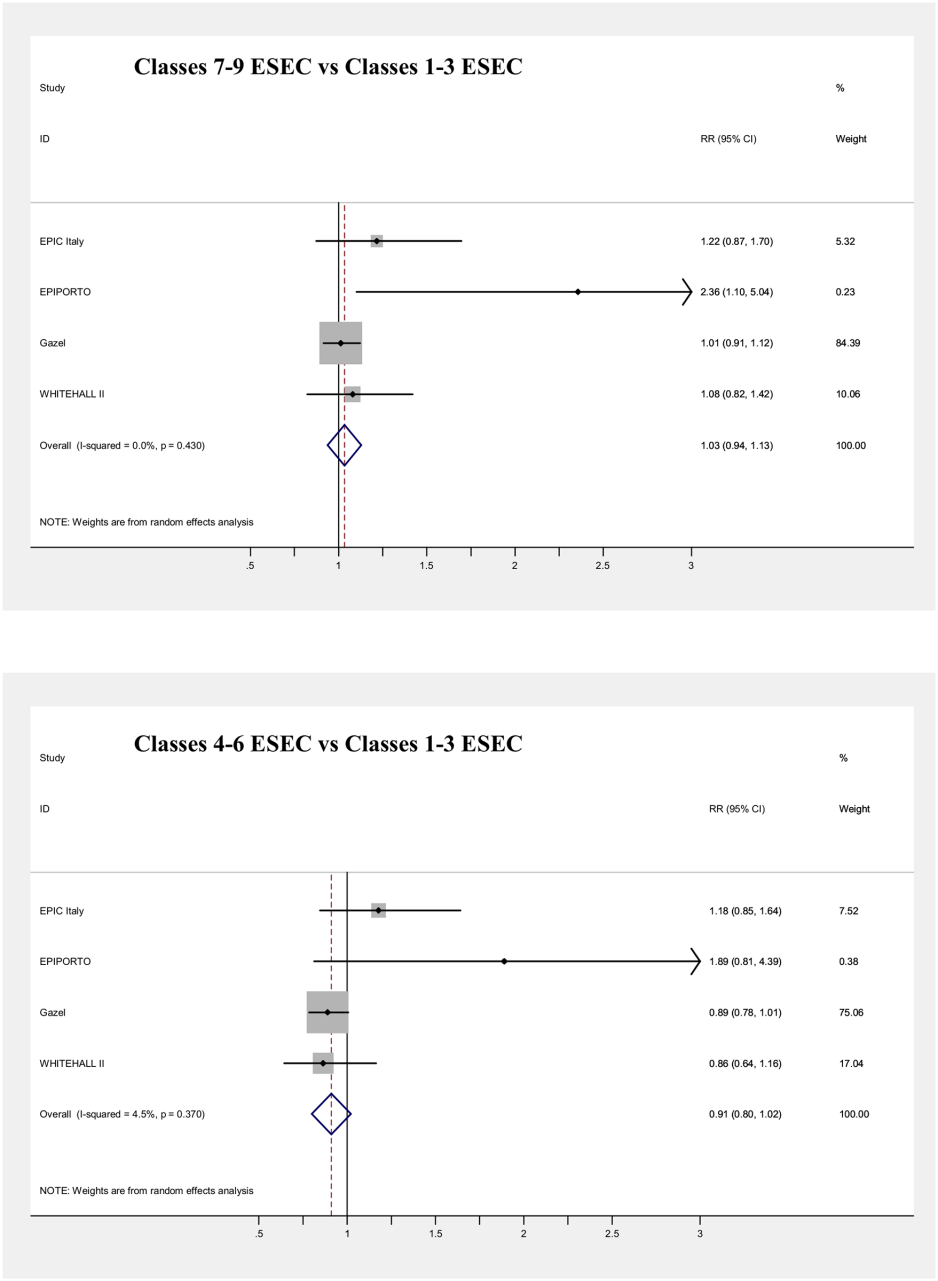
Meta-analysis of the association between father’s job and mortality (Males).

**Fig 8:**
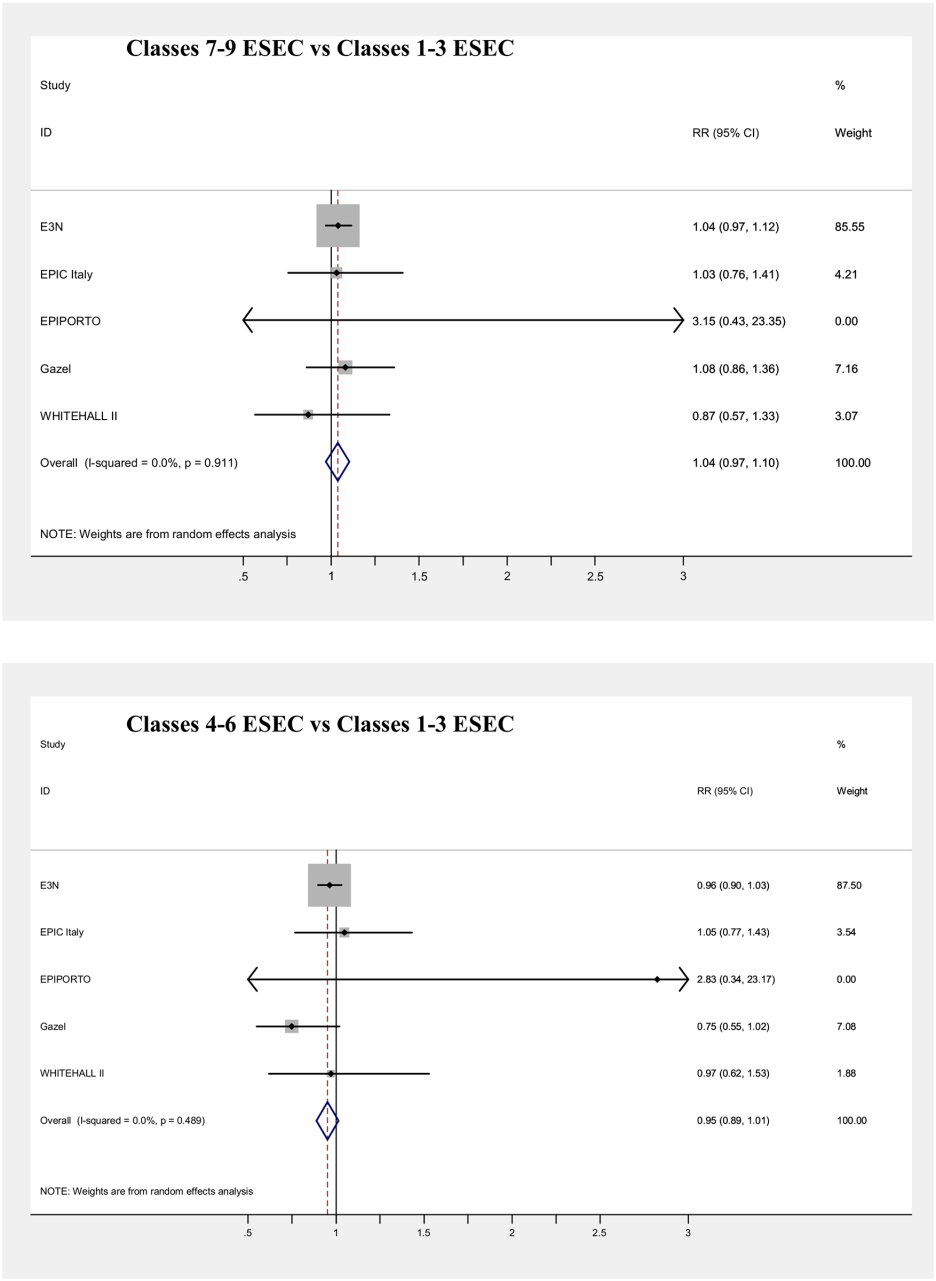
Meta-analysis of the association between father’s job and mortality (Females).

Main results of the meta-analyses on the associations between SEP indicators and mortality (adjusted for age) are presented in Table 6 and in Figs 3, 4, 5, 6, 7 and 8, for men and women separately. Detailed results for each cohort are available in the S1 and S2 Tables, for men and women respectively.

**Table 6:** Results of the association between socioeconomic factors and mortality from random effect meta-analysis.

	Male			Female		
	RR	95% CI	I^2^	RR	95% CI	I^2^
**Education**						
**Three levels variable**						
*primary or lower secondary school*	1.36	1.17–1.55	72.00%	1.15	1.05–1.25	34.70%
*higher secondary school*	1.22	1.07–1.37	45.60%	1.01	0.97–1.05	0.00%
*tertiary education*	Ref			Ref		
**Four levels variable**						
*primary or lower secondary school*	1.70	1.49–1.91	0.00%	1.19	1.04–1.35	31.70%
*vocational school*	1.47	1.30–1.64	0.00%	1.06	0.99–1.14	0.00%
*higher secondary school*	1.33	1.13–1.53	0.00%	0.99	0.95–1.03	0.00%
*tertiary education*	Ref			Ref		
**Current job**						
**Two levels variable**						
*Manual workers*	1.40	1.09–1.71	75.40%	1.05	0.93–1.17	0.00%
*Non manual workers*	Ref			Ref		
**Three levels variable**						
*Classes 1–3 ESEC*	Ref			Ref		
*Classes 4–6 ESEC*	1.37	1.25–1.48	0.00%	1.06	1.00–1.13	0.00%
*Classes 7–9 ESEC*	1.84	1.66–2.04	0.00%	1.14	1.06–1.22	0.00%
**Current/last job**						
**Two levels variable**						
*Manual workers*	1.37	1.19–1.56	83.00%	1.05	0.99–1.12	0.00%
*Non manual workers*	Ref			Ref		
**Three levels variable**						
*Classes 1–3 ESEC*	Ref			Ref		
*Classes 4–6 ESEC*	1.41	1.26–1.57	53.70%	1.11	0.91–1.31	0.00%
*Classes 7–9 ESEC*	1.81	1.61–2.01	47.60%	1.16	0.95–1.37	0.00%
**Father's job**						
**Two levels variable**						
*Manual workers*	1.01	0.93–1.08	0.00%	1.00	0.96–1.05	0.00%
*Non manual workers*	Ref			Ref		
**Three levels variable**						
*Classes 1–3 ESEC*	Ref			Ref		
*Classes 4–6 ESEC*	0.91	0.80–1.02	4.50%	0.95	0.89–1.01	0.00%
*Classes 7–9 ESEC*	1.03	0.94–1.13	0.00%	1.04	0.97–1.10	0.00%

All SEP indicators, except fathers’ occupational class, were associated with mortality in men, while only education and current occupational class showed an association in women.

Statistically significant increased risks of mortality were found in men with high secondary school (RR = 1.22, I^2^ = 45.6%) and with primary or lower secondary school (RR = 1.36), compared to men with tertiary education (Fig 3), although with a significant heterogeneity among cohorts for the latter risk estimate (I^2^ = 72.0%). In women, only participants with the lowest level of education showed a significant increase in risk, compared to the highest one (RR = 1.15), whereas among those with intermediate education the risk was close to one (RR = 1.01). A clear trend of increase in risk was also observed among men, but not among women, when subjects who attended a vocational school were separated by those in the lowest educational class, in the cohorts for which this information was available.

The strongest effect was observed for occupational class based on current/last job in men, with an 81% increase in mortality risk, comparing the lowest with the highest class, and a 41% increase in the intermediate one, both of which were statistically significant (Fig 5). Pooled risk estimates were quite similar when only the employed population was considered and was categorized through the current job, or also retired or unemployed subjects who worked in the past were included, classifying them using the last job held. In contrast, among women both lower occupational categories displayed a modest significant increase in risk (RR = 1.06 and RR = 1.14 for the intermediate and the low occupational classes, respectively), which however lost significance when the population of individuals formerly employed was included in the analyses (Fig 6). A low level of heterogeneity was present among risk estimates across studies for occupational class in both genders.

When the association between occupational class and mortality was examined keeping separate low-grade non-manual workers and skilled workers (ESEC classes 7 and 8) from semi-skilled and unskilled workers (ESEC class 9), we found in men a RR = 1.66 (95% CI 1.29–2.02) for low-grade non-manual workers and skilled workers vs. the highest occupational class. For semi-skilled and unskilled workers the risk was slightly higher (RR = 1.76, 95% CI: 1.29–2.30), although with a wide overlap between confidence intervals of the risk estimates (S1 Fig). Among women, the mortality risk was equal to one for low-grade non-manual workers and skilled workers, compared to the highest occupational class, while it was slightly increased for semi-skilled and unskilled workers, but also with great uncertainty of the point estimate (RR = 1.22, 95% CI: 0.63–1.80).

Null results in the pooled analyses were observed both in men and women for the type of occupation of subjects’ fathers (Figs 7 and 8). Most of the mortality risks were around unity, except for the Epiporto and EPIC-Italy cohorts. In the first one, in both genders low or intermediate father’s occupational classes were associated with mortality risks around 2–3 times higher than the highest class, although only the risk estimate for men with fathers in the lowest class reached statistical significance (RR = 2.36). Risks were also non-significantly elevated among men in the EPIC-Italy cohort (RR = 1.22 and RR = 1.18 for the lowest and the intermediate categories, respectively). Even when the category of semi-skilled and unskilled workers was examined separately from the rest of the lowest occupational class, the results for this category were in each cohort similar to those obtained using the three-level classification and the pooled risk of mortality was also around unity in both genders (S2 Fig). However, most cohorts with information on father’s occupation included quite few deaths, with the consequence of low statistical power and risk estimates characterized by wide confidence intervals. Moreover, for both genders the results of the meta-analysis were driven by one French occupational cohort (more than 80% of the weight for Gazel in men and for E3N in women).

Low personal income was associated with an increased risk of mortality, but among men risk estimates were very different in the two cohorts examined (RR = 3.60 and RR = 1.35 for the lowest vs. the highest quintile in CoLaus/PsycoLaus and WHIP-retired, respectively), whereas among females no deaths occurred in the reference category in one study (CoLaus/PsycoLaus) and in the other one the risk in the lowest quintile was similar to that of males (RR = 1.33). No meta-analysis was performed on income and mortality, considering that the meta-RR for men would have been strongly influenced by one study (WHIP-retired), because of its size.

## Discussion

The objectives of this study were: firstly, to harmonize the SEP classifications on occupational class, education and income among the cohorts belonging to the LIFEPATH Consortium and to present their distribution in the different cohorts; and, secondly, to compare social gradients in overall mortality observed in the available cohorts according to the different SEP indicators.

In spite of differences in recruitment among the cohorts, especially in terms of time, age, gender composition and type of sample, variability in the distribution of the socioeconomic indicators in the different study populations was relatively low.

Regarding education, in most cohorts the proportion of subjects in the lowest category was consistently around or above 50%, with the exception of E3N, where the proportion was very low because it was composed mainly of teachers, Constance, which started only recently and includes volunteering subjects younger than in other cohorts, and Whitehall II, which includes a large proportion of male high-grade employees. Data are more variable across cohorts for the proportion of subjects with tertiary education, which however seems to reflect mostly cross-country differences in educational achievements and in school systems.

The distribution of occupational class, based on current/last job, was also quite consistent across cohorts, except for E3N and Constances, and for Gazel and Whitehall II, where the proportion of subjects in higher occupational classes was higher, in spite their educational level was similar to other cohorts; a likely explanation is that these workers were employed in public or semi-public work organizations where career advancements were more linked to skills acquired at the workplace rather than to formal education.

Despite differences in the study populations and in the SEP categories employed, their distribution in the different cohorts was roughly comparable to that observed in other studies conducted in Europe, including several performed on representative samples of the general population, both for education [5,63–65] and occupational class [3,5,8,65].

A shift toward higher occupational classes was noted between fathers’ and subjects’ occupations in most cohorts, which seems to reflect an increasing trend of social improvement in these populations, as well as the decrease of the proportion of people employed in manual jobs and in agriculture during the last fifty years. As expected, the SEP indicators examined were quite correlated each other, especially occupational class and education, although with some differences between cohorts; the lowest correlation was observed for the E3N and the Gazel cohorts, for the reasons explained above.

The association between SEP and mortality strongly differed by gender, with much higher and significant associations in men than women, although characterized by variable strength in the different cohorts.

Regarding education, mortality risks were significantly heterogeneous among men in the lowest category, so that the increases computed through the meta-analysis cannot be considered a reliable pooled estimate (36%), whereas no significant heterogeneity was present for the intermediate category, resulting in a 22% increased risk compared to the most educated. These figures appear comparable to those produced in other studies on education and mortality conducted in Europe and U.S. for men, indicating that the predictive validity of our education classification was relatively good [5,14,41,63,64,66]. Among women, the association with education was instead lower than that observed in most other studies [5,13,41,65,67], although it was in the expected direction. Gradients in mortality by educational level were possibly underestimated by 5–15% because of the aggregation of low secondary and primary school, assuming that the two groups were in most countries of similar size; in fact, those studies where the two categories were kept separate have shown in general 10–30% higher mortality in the primary education category, compared to low secondary one [5; 63–65]. Nonetheless, the three-level educational classification adopted has been widely used in other European studies [68,69], also because it allows aggregating subjects who went through different educational systems with different length of compulsory education.

For occupational class, our mortality results among men are generally in agreement with those observed in other European countries, considering differences in the categories used in this, compared to other studies [3,5,12,65]. The choice of having more robust categories, keeping aggregated ESeC classes 7, 8 and 9 (low grade non-manual, skilled manual and unskilled manual workers), may have led to an underestimation of the actual occupational social gradient, likely of magnitude similar to that for education, considering the relative proportions of these workers’ groups and the differences in mortality observed between them in other studies [3,5,12,28,65,70]. Again, the small and non-significant occupational gradient in mortality found among women seems in contrast with the higher risks observed in several other studies investigating mortality [5,65,71–75] or morbidity [7,76,77], although in most studies the gradient was lower than among men and in some others no or only slight increases in risk were found [70,78–81]. The shallower social gradient observed among women, compared to other reports in the literature, appears difficult to explain and may be related to specific features of some of the cohorts examined (e.g. lower participation of ill women belonging to lower social strata), although risk estimates were quite homogeneous across them.

In the two studies with information on income, participants in the lowest income quintiles showed a significantly increased risk of mortality, although very different in. Such a difference appears due to the fact that in the WHIP-retired cohort, workers with the highest salary were the executive, who represented only 5% of the total population, so the highest quintile included also other three quarters of white collars with a lower income. These results indicate that use of quantiles is not appropriate to describe income distribution, as subjects within quantiles may be too heterogeneous to be pooled together, but it would be preferable to put limits between categories in correspondence of major discontinuities in income distribution. Reports in the literature are consistent in showing an association with income, but with a wide variability in the strength of the association observed [41,65,66,79]. Our conclusion is that available data on income and mortality were too scarce to conduct a meaningful examination of their predictive validity.

Unexpectedly, no association was found between father’s occupational class and mortality, except for the lowest category in Epiporto. This appears in contrast with consistent reports of increased mortality among subjects with childhood disadvantage, measured through a variety of social indicators [3,38,39,71,82], although with moderate increases in risk, generally not exceeding 50%. The lack of an association in our study cannot be explained by the choice of aggregating ESEC classes 7, 8 and 9 together, because even keeping separate subjects whose father was in the lowest ESEC class, their pooled risk of mortality was still around one, compared to people whose father was in the highest class. The null results of this meta-analysis are possibly due to the specificity of the two French cohorts, both occupational-based, which drive the results because of their large weight. Unfortunately, we could not investigate the association with other SEP measures in childhood, because of data unavailability.

In general, results on the relationship between our classification of occupational class and mortality showed a more consistent relationship across studies than the other social measures. They are also in agreement with the conclusions of an article on morbidity and occupational class, measured through the EG scheme, by the European Network on Social Inequalities [31]. Another source of support to our occupational class definition comes from the work by Evans and Mills (2000) [83], who examined the criterion validity of the EG scheme on British survey data from 1996, using eight indicators of the employment relationships and applying to them latent class analysis; these authors also found three latent classes, which corresponded to service, intermediate and labor contract occupations, supporting this way a good criterion validity of a three-category classification.

Among the strengths of the study, the data employed derive from many studies participating in a large consortium, which together includes a population of over 500,000 subjects. The harmonization of the socioeconomic indicators available in the different cohorts and the large size of the study allowed us to assess with a large degree of statistical power differences in mortality by SEP according to the different indicators. Moreover, the availability of such a large amount of harmonized data combined with that of many biological measurements at different times during the lifecourse, will provide a unique opportunity to try identifying mechanisms and pathways leading from low SEP to unhealthy ageing.

Regarding limitations, as said in the discussion with respect to both education and occupational class, the wide categories used have likely produced a certain degree of heterogeneity within the categories, whose consequence would be an underestimation of the true social gradient in mortality.

However, from results of our own analyses and those in the literature, such an underestimation appears quite small and counterbalanced by the advantage of having robust social categories, each with large numbers of events, as well as avoiding the misclassification bias potentially present in the effort of assigning subjects to more detailed and extended categories. The heterogeneous nature of the cohorts may also be a limitation, particularly the presence of both population-based studies and occupational studies in the analysis on occupational class. In mitigation, it could be argued that, this provided greater statistical power to evaluate the relation between SEP and mortality. The choice of using a measure of occupational class based on employment relations, rather than prestige, may have also led to an underestimation of the true social gradient in mortality, as suggested by the results of two British studies [70,81]. This could be because prestige-based indicators are more closely linked to household income availability, education, or lifestyle than those constructed on employment relations. A measure based on employment relations may also be an inferior indicator of social and economic conditions in women in nations where they have a weaker attachment to the labour market.

In conclusion, among men the proposed three-level classifications of occupational class and education appear to not differ substantially from more detailed classifications in discriminating between main social strata, and in predicting differences in mortality between them, which however seem to be slightly underestimated compared to other studies. In contrast, among women mortality was only moderately increased in the lowest categories of education and current occupational class, possibly because classifying them through their sole occupation, without taking into account the SEP of their partners or of their household, may imply a misclassification of their social position leading to attenuation of differences in health outcomes. The lack of association with father’s occupational class was unexpected: possibly attributable to a “French cohort effect”, due to the occupational nature of the Gazel and E3N cohorts, which drive strongly the results, although the higher risks found in Portugal and Italy suggest that this dimension may be more relevant for Southern Europe.

## Supporting information

S1 File Cohorts description. (DOC)

S2 File Harmonization of EDUCATION. (DOC)

S3 File Harmonization of CURRENT/LAST JOB. (DOC)

S4 File Harmonization of FATHER’S JOB. (DOC)

S1 Table Association between socioeconomic variables and mortality separated by cohort. Males.
(DOC)

S2 Table Association between socioeconomic variables and mortality separated by cohort. Females.
(DOC)

S1 Fig Meta-analysis of the association between current/last job and mortality separating skilled and semi- and unskilled workers. (DOC)

S2 Fig Meta-analysis of the association between fathers’ job and mortality separating skilled and semi- and unskilled workers. (DOC)
