# Supplementary material for: Socioeconomic indicators in epidemiologic research: A practical example from the LIFEPATH study
Source: PLoS One. 2017 May 30;12(5):e0178071. doi: 10.1371/journal.pone.0178071 (PMC5448763; doi:10.1371/journal.pone.0178071)
Supplement: S1 File — (DOC) [file pone.0178071.s001.doc]

# **S1 File. Cohorts description (information about ethical approval of each study is provided in italic)**

**COLAUS.** The CoLaus (COhorte LAUSannoise) is an ongoing prospective study assessing the clinical, biological and genetic determinants of cardiovascular disease in the city of Lausanne, Switzerland (Firmann et al., 2008). The initial survey was conducted between 2003 and 2006 and included 6733 participants aged between 35 and 75 years; the first follow-up survey was conducted 5.5 years afterwards and included 5064 participants. In each survey, data on socio-economic status, lifestyle, mental status and cardiovascular risk factors is collected by questionnaire or clinical examination.

*The Study was approved by the Institutional Ethic's Committee of the University of Lausanne*

**CONSTANCES.** The CONSTANCES cohort was established in late 2012 (Zins et al., 2015). It was designed as a randomly selected representative sample of French adults aged 18-69 years at inception; 200,000 subjects will be included over a five-year period. At enrolment the participants fill questionnaires collecting data on health, lifestyle, individual, familial, social and occupational factors, and life events and benefits from a comprehensive health examination. The follow-up includes a yearly self-administered questionnaire, a health examination every 5 years and an annual linkage to social and health national databases.

*All confidentiality, safety and security procedures were approved by the French legal authorities. According to the French regulations, the CONSTANCES Cohort project has obtained the authorization of the National Data Protection Authority (*Commission nationale de l’informatique et des libertés*—CNIL). In addition, CONSTANCES was approved by the National Council for Statistical Information (*Conseil national de l’information statistique*—CNIS), the National Medical Council (*Conseil national de l’Ordre des médecins*—CNOM), the Institutional Review Board of the National Institute for Medical Research-INSERM and our local Committee for Persons Protection (*Comité de protection des personnes*).*

**E3N.** The E3N study (Étude Epidémiologique auprès de femmes de la Mutuelle Générale de l’Education Nationale) is a prospective cohort of 98,995 women aged 40-65 years at recruitment in 1990 and it constitutes the French component of EPIC. The study was established to produce a large mass of data relevant for the identification of environmental and molecular causes of cancer and other chronic diseases, and to contribute to the development of effective public health strategies. Data on residence, education, early life events, exposures, lifestyle factors and life events were collected at baseline and follow-up questionnaires were sent every 2-3 years with more than 80% response. Mortality data were obtained by means of regular record linkage with various French mortality databases.

*The study was approved by the French National Commission for Computed Data and Individual Freedom (Commission Nationale Informatique et Libertés), ethical clearance was also obtained from INSERN Institutional Review Board for the use of biospecimens.*

**EPIC – Italy.** The European Prospective Investigation into Cancer and Nutrition (EPIC) is a large European study on diet and cancer. The Italian component of EPIC EPIC-Italy) recruited 47,749 adult volunteers (men and women) at five centres: Varese and Turin in northern Italy, Florence in central Italy and Naples and Ragusa in southern Italy. All participants signed an informed consent form and completed two questionnaires: one about dietary habits (food-frequency) and one about lifestyle, with information on education, socioeconomic status, occupation, history of previous illnesses and surgery, lifetime tobacco use and alcohol consumption and physical activity. EPIC database records were linked to cancer and regional mortality registries after EPIC database quality control. All EPIC-Italy centers except Naples are covered by population-based cancer registries. In Naples, follow-up information was collected from electronic hospital discharge records and also by periodic personal contact with participants.We are including in Lifepath centres from EPIC Italy (N=34,148) with the exclusion of Florence.

*The study was approved by the ethical review boards of the International Agency for Research on Cancer, and of the collaborating institutions responsible for subject recruitment in each of the EPIC recruitment centres.*

**EPIPORTO.** The EPIPorto is a general adult population-based cohort established with the initial aim of evaluating the major determinants of cardiovascular health. For this purpose, 2485 (949 men and 1536 women) adult dwellers in Porto, aged 18 years or over, were recruited between 1999 and 2003 using random digit dialling. At all waves, information was collected using questionnaires administered by trained interviewers, self-administered questionnaires and objective measurements were made, including physical examination and blood tests (Ramos et al, 2004; Pereira et al, 2012).

*The local institutional ethics committee (city of Porto) approved the study and all participants gave written informed consent.*

**GAZEL.** The GAZEL study was established in 1989 among employees of the French national gas and electricity company, Electricité de France-Gaz de France (EDF-GDF) (Goldberg et al., 2007, 2015). At baseline (1989), 20,625 employees (15,011 men and 5,614 women), aged 35–50, gave consent to participate. The study design consists of an annual questionnaire used to collect data on health, lifestyle, individual, familial, social and occupational factors, and life events.

*The study was approved by the French authority for data confidentiality (Commission Nationale Informatique et Liberté) and by the Ethics Evaluation Committee of the Institut National de la Santé et de la Recherche Médicale (INSERM).*

**MCCS.** The Melbourne Collaborative Cohort Study is a prospective cohort study of 41 514 participants (24 469 women) living in Melbourne, Australia. Caucasian volunteers aged between 40 and 69 years were recruited in randomly selected census districts. At baseline (1990–1994), participants attended clinics where demographic, lifestyle and dietary information were collected and anthropometric measurements were performed.

*The Cancer Council Victoria's Human Research Ethics Committee approved the study protocol.*

**SKIPOGH**. The Swiss Kidney Project on Genes in Hypertension is a longitudinal family-based study, following the standardised EPOGH (European Project on Genes in Hypertension) protocol. Baseline examination was conducted between 2009 and 2013. Three-year follow-up examination started in 2013 and is currently ongoing (expected to be finished in 2015). The aim of the study is to explore the role of genes and kidney haemodynamics in blood pressure regulation and kidney function in the general population. From December 2009 to March 2013, adult participants were recruited in two regions (Berne and Geneva) and one city (Lausanne) of Switzerland. A random sample of the inhabitants was drawn using different strategies. Inclusion criteria were (1) having a minimum age of 18 years; (2) being of European ancestry; (3) having ≥one and ideally three first-degree family members willing to participate; and (4) providing a written informed consent. Pregnant or breastfeeding women were not included. The general participation rate was 27.1%. At baseline, we collected data on cardiovascular and metabolic risk factors as well as on the prevalence of kidney and cardiovascular diseases. During follow-up, we are collecting data on new kidney and cardiovascular events. The primary endpoints are fatal and non-fatal strokes, ischaemic heart disease, heart failure and chronic kidney disease. The 1128 participants (537 men and 591 women) belong to 272 nuclear families.

*The SKIPOGH study was approved by the Human Research Ethics Committee, Lausanne University Hospital and University of Lausanne (Lausanne, Switzerland), by the Ethics Committee for the Research on Human Beings, Geneva University Hospitals (Geneva, Switzerland), and by the Ethics Committee of the Canton of Bern, (Bern, Switzerland).*

**TILDA.** The Irish Longitudinal Study on Ageing (TILDA) is a large prospective cohort study examining the social, economic, and health circumstances of 8,175 community-dwelling older adults (3744 men, 4431 women) aged 50 years and older at the time of recruitment and resident in the Republic of Ireland. Baseline examination (phase 1) took place during 2009-2011 and participants are followed up biennially. There were three components to the survey. Respondents completed a computer-assisted personal interview (n = 8,175) and a separate self-completion paper and pencil module (n = 6,915) which collected information that was considered sensitive. All participants were invited to undergo a separate health assessment at one of two national centers using trained nursing staff (n=5897). A total of 5036 respondents completed the health-centre based assessment and a further 861 respondents completed a home-based assessment which involved a reduced set of tests. A more detailed exposition of study design, sample selection and protocol is available elsewhere (Whelan & Savva, 2013).

*Ethical approval for the study was obtained from the Trinity College Research Ethics committee.*

**WHIP.** The Work History Panel is based on a sample of individual-level data from the Social Security Administration archives in Italy, covering almost 8% of all Italian workers employed in the private sector in 1985-2010. Unlike all other cohorts in LIFEPATH, it does not have biological samples, but it provides very rich information on income, pensions, unemployment benefits, disability indemnities, workplace and job contracts, linked to hospital and mortality follow-up.

*All activities, regardless of their complexity or depth, were conducted in accordance with Italian regulations on privacy and with the approval of the national institutes involved. In 2013, the WHIP-Salute database has been included in the Italian National Statistics Program under responsibility of the Ministry of Health. According to Italian regulation on privacy, it provides for microdata files for research purposes, following the presentation of a research protocol.*

**WHITEHALL-II**. **﻿**The Whitehall II study was established in 1985 to examine the socioeconomic gradient in health among 10,308 London-based civil servants (6,895 men and 3,413 women) aged 35–55. Baseline examination (phase 1) took place during 1985–1988, and involved a clinical examination and a self-administered questionnaire containing sections on demographic characteristics, health, lifestyle factors, work characteristics, social support, and life events.

*The University College London ethics committee approved this study.*
