# Supplementary material for: Socioeconomic indicators in epidemiologic research: A practical example from the LIFEPATH study
Source: PLoS One. 2017 May 30;12(5):e0178071. doi: 10.1371/journal.pone.0178071 (PMC5448763; doi:10.1371/journal.pone.0178071)
Supplement: S2 File — (DOC) [file pone.0178071.s002.doc]

**S2 File. Harmonization of EDUCATION**

**Three levels variable**

edulev_3=1 🡪 primary or lower secondary school

edulev_3=2 🡪 higher secondary school= 2

edulev_3=3 🡪 tertiary education (post-secondary)

| **Colaus** | Edulev_3=1 | Apprenticeship (apprentissage)  Compulsory education (scolarité obligatoire) |
| --- | --- | --- |
|  | Edulev_3=2 | High school (baccalauréat, maturité) |
|  | Edulev_3=3 | Postgraduate education (maîtrise, diplôme supérieur (technicum, etc..)  University education (université, hautes écoles) |
| **Constances** | Edulev_3=1 | Primary or lower secondary  Vocational school |
|  | Edulev_3=2 | Higher secondary school |
|  | Edulev_3=3 | Tertiary education |
| **E3N** | Edulev_3=1 | No education  Certificate of Primary Study |
|  | Edulev_3=2 | BTEC First Diploma - Youth Training  A levels to A levels + 2 years |
|  | Edulev_3=3 | A levels + 3 years to A levels + 4 years  At Minimum A levels + 5 years |
| **EPIC Italy** | Edulev_3=1 | None (nessuna)  Primary School (licenza elementare)  Lower secondary school (licenza media)  Vocational school (scuola professionale) |
|  | Edulev_3=2 | Higher secondary school (diploma/maturità) |
|  | Edulev_3=3 | BSc (corso parauniversitario)  MSc (laurea) |
| **EPIPORTO** | Edulev_3=1 | Less or equal 9 year of school |
|  | Edulev_3=2 | Between 10 and 12 years of school |
|  | Edulev_3=3 | More than 12 years of school |
| **GAZEL** | Edulev_3=1 | Basic education certificate (certificat d’étude primaire)  Junior secondary education certificate (BEPC)  Certificate of professional competence (CAP)  Vocational certificate (BEP, BP, BEC, BEI) |
|  | Edulev_3=2 | Baccalaureate (baccalauréat)  Other diploma (autre diplôme) |
|  | Edulev_3=3 | Undergraduate degree (supérieur technique de niveau BTS, DUT)  Other academic degree (autre enseignement supérieur enseignement ) |
| **MCCS** | Edulev_3=1 | ’Never attended school  Some primary school  Completed primary school  Some high/technical school  Other qualification (eg. Trade certificate) |
|  | Edulev_3=2 | Completed high/technical school |
|  | Edulev_3=3 | Completed tertiary degree or diploma  Some study towards a tertiary degree or diploma |
| **SKIPOGH** | Edulev_3=1 | No diploma  Mandatory school  General secondary training, level (trading school (sellers), diploma)  Secondary vocational training inferior level (Federal Certificate of Capacity) |
|  | Edulev_3=2 | Secondary training preparing for higher studies  Secondary vocational training, superior level  Superior non-university training, 3 years or less  Superior non-university training, more than 3 years |
|  | Edulev_3=3 | University, Technical university (ETH) |
| **TILDA** | Edulev_3=1 | None  Some primary (not complete)  Primary or equivalent  Intermediate/junior/group certificate or equivalent |
|  | Edulev_3=2 | Leaving certificate or equivalent  Diploma/certificate |
|  | Edulev_3=3 | Primary degree  Postgraduate/higher degree |
| **WHITEHALL II** | Edulev_3=1 | No academic qualification  School certificate  `O' level |
|  | Edulev_3=2 | Matriculation  'A' level, SCE higher  'S' level  City and Guilds  National diplomas or certificates (eg: ONC, HND, ) |
|  | Edulev_3=3 | BA/Bsc  University or CNAA Higher degree (MA/MSc, PhD) |

**Four levels variable**

edulev_4=1 🡪 primary or lower secondary school

edulev_4=2 🡪 vocational school

edulev_4=3 🡪 higher secondary school

edulev_4=4 🡪 tertiary education (post-secondary)

| **Colaus** | Edulev_4=1 | Compulsory education (scolarité obligatoire) |
| --- | --- | --- |
|  | Edulev_4=2 | Apprenticeship (apprentissage) |
|  | Edulev_4=3 | High school (baccalauréat, maturité) |
|  | Edulev_4=4 | Postgraduate education (maîtrise, diplôme supérieur (technicum, etc..)  University education (université, hautes écoles) |
| **Constances** | Edulev_4=1 | Primary or lower secondary |
|  | Edulev_4=2 | Vocational school |
|  | Edulev_4=3 | Higher secondary school |
|  | Edulev_4=4 | Tertiary education |
| **E3N** | Edulev_4=1 | No education  Certificate of Primary Study |
|  | Edulev_4=2 | BTEC First Diploma - Youth Training |
|  | Edulev_4=3 | A levels to A levels + 2 years |
|  | Edulev_4=4 | A levels + 3 years to A levels + 4 years  At Minimum A levels + 5 years |
| **EPIC Italy** | Edulev_4=1 | None (nessuna)  Primary School (licenza elementare)  Lower secondary school (licenza media) |
|  | Edulev_4=2 | Vocational school (scuola professionale) |
|  | Edulev_4=3 | Higher secondary school (diploma/maturità) |
|  | Edulev_4=4 | BSc (corso parauniversitario)  MSc (laurea) |
| **GAZEL** | Edulev_4=1 | Basic education certificate (certificat d’étude primaire)  Junior secondary education certificate (BEPC) |
|  | Edulev_4=2 | Certificate of professional competence (CAP)  Vocational certificate (BEP, BP, BEC, BEI) |
|  | Edulev_4=3 | Baccalaureate (baccalauréat)  Other diploma (autre diplôme) |
|  | Edulev_4=4 | Undergraduate degree (supérieur technique de niveau BTS, DUT)  Other academic degree (autre enseignement supérieur enseignement ) |
| **SKIPOGH** | Edulev_4=1 | General secondary training, level (trading school (sellers), diploma)  Secondary vocational training inferior level (Federal Certificate of Capacity) |
|  | Edulev_4=2 | No diploma  Mandatory school |
|  | Edulev_4=3 | Secondary training preparing for higher studies  Secondary vocational training, superior level  Superior non-university training, 3 years or less  Superior non-university training, more than 3 years |
|  | Edulev_4=4 | University, Technical university (ETH) |
