# Supplementary material for: Socioeconomic indicators in epidemiologic research: A practical example from the LIFEPATH study
Source: PLoS One. 2017 May 30;12(5):e0178071. doi: 10.1371/journal.pone.0178071 (PMC5448763; doi:10.1371/journal.pone.0178071)
Supplement: S3 File — (DOC) [file pone.0178071.s003.doc]

**S3 File. Harmonization of CURRENT/LAST JOB**

**Two levels variable**

occ_stat_2/occ_last_2=1 🡪 manual workers

occ_stat_2/occ_last_2=2 🡪 non manual workers

| **Colaus** | Occ_stat_2=1 | Employee unskilled  Farmer  Maneuver  Qualified worker |
| --- | --- | --- |
|  | Occ_stat_2=2 | Qualified worker  Independent worker  Senior manager  Middle management  Professional (Profession libérale)  Entrepreneur |
| **Constances** | Occ_stat_2=1 | Farmer  Worker |
|  | Occ_stat_2=2 | Employer  Manager, intellectual profession  Middle profession  Clerical or service employee |
| **E3N** | Occ_stat_2=1 | Ouvrier contremaître  Agriculteur exploitant  Artisan  Contremaître - Agent de maîtrise  Personnel des services directs aux particuliers  Ouvrier qualifié  Ouvrier non qualifié  Ouvrier agricole  CNAS |
|  | Occ_stat_2=2 | Directeur d'école 1er degré  Instituteur  Agrégé  Certifié  Adjoint d'enseignement  PEGC  Professeur de lycée professionnel  Maître auxiliaire  Professeur d'Université  Maître de conférence des Universités  Direction - Inspection  Chef d'établissement 2nd degré  Attaché  Conseiller d'éducation  Secrétaire  Infirmier( e)  Commis, agent ou adjoint administratif  Sténo-dactylo  Agent de bureau  Agent d'établissement d'enseignement  Autre : en clair NOENSAUT_CAR  Commerçant et assimilé  Chef d'entreprise (10 salariés ou +)  Profession libérale  Cadre fonction publique, profession intellectuelle, artistique  Cadre d'entreprise, profession intermédiaire de la santé, fonction publique et assimilé  Profession intermédiaire administrative et commerciale des entreprises  Technicien  Employé de la fonction publique  Employé administratif d'entreprise  Employé de commerce |
| **EPIC Italy** | Occ_stat_2=1 | farmer  unskilled workers  skilled workers |
|  | Occ_stat_2=2 | retailer  clerical worker  professionals and managers |
| **EPIPORTO** | Occ_stat_2=1 | Skilled agricultural and fishery workers  Semi-skilled occupations  Unskilled occupations |
|  | Occ_stat_2=2 | Higher Professions/occupations  Intermediate Professions/occupations  Non-manual skilled occupations |
| **GAZEL** | Occ_stat_2=1 | Labourer  Employee |
|  | Occ_stat_2=2 | Administrative executive  Technical executive  Administrative supervisor  Technical supervisor  Sales representative  Patrolman, block manager  Teacher, school staff  Trainee agent |
| **SKIPOGH** | Occ_stat_2=1 | Skilled manual worker (metalworker, mechanic, plumber, miner,…)  Unskilled manual worker (carrier, driver, cleaner)  Farmer |
|  | Occ_stat_2=2 | Self-employed, liberal professions (doctors, lawyers,…)  Self-employed, commerce and trade (butcher, shoemaker, hairdresser,…)  Higher level managers (directors, managers,…)  Executive managers (engineer, magistrate,…)  Skilled non-manual worker (secretary, technician, programmer,…)  Unskilled non-manual worker (waiter, postman, clerk,…)  Skilled workers in the health service (nurse, physiotherapist, laboratory worker,…)  Teachers, educators  Professors, headmasters  Clergy  Public safety, public force (military, policemen, firemen) |
| **TILDA** | Occ_stat_2=1 | Skilled manual  Semi-skilled  Unskilled  Farmers |
|  | Occ_stat_2=2 | Professional workers  Managerial & technical  Non-manual |
| **WHIP-retired** | Occ_stat_2=1 | Apprentice  Laborer |
|  | Occ_stat_2=2 | Employee  Supervisor  Manager |
| **Whitehall II** | Occ_stat_2=1 | Clerical and Support Staff, e.g. messengers, porters, telephonists, typists at right wave |
|  | Occ_stat_2=2 | Unified Grade 1–6 at right wave  Unified Grade 7 at right wave  Senior Executive Officer at right wave  Higher Executive Officer at right wave  Executive Officer at right wave |

**Three levels variable**

occ_stat_3/occ_last_3=1 🡪 higher professionals and managers, lower professionals and managers; higher clerical, services and sales workers (Class 1-3 ESEC – European Socio-economic Classification – 9 classes)

occ_stat_3/ occ_last_3=2 🡪 small employers and self-employed; farmers; lower supervisors and technicians (Class 4, 5, and 6 ESEC) –include here intermediate professions such as teachers, nurses, etc

occ_stat_3/ occ_last_3=3 🡪 lower clerical, services, and sales workers; skilled workers; semi – and unskilled workers (Class 7-9 ESEC)

| **Colaus** | Occ_stat_3=1 | Senior manager  Professional (Profession libérale)  Entrepreneur |
| --- | --- | --- |
|  | Occ_stat_3=2 | Farmer  Independent worker  Middle management |
|  | Occ_stat_3=3 | Employee unskilled  Qualified worker  Maneuver  Qualified worker |
| **Constances** | Occ_stat_3=1 | Manager, intellectual profession |
|  | Occ_stat_3=2 | Farmer  Employer  Middle profession |
|  | Occ_stat_3=3 | Clerical or service employee  Worker |
| **E3N** | Occ_stat_3=1 | Directeur d'école 1er degré  Maître auxiliaire  Professeur d'Université  Maître de conférence des Universités  Direction - Inspection  Chef d'établissement 2nd degré  Chef d'entreprise (10 salariés ou +)  Profession libérale  Cadre fonction publique, profession intellectuelle, artistique |
|  | Occ_stat_3=2 | Instituteur  Agrégé  Certifié  Adjoint d'enseignement  PEGC  Professeur de lycée professionnel  Infirmier( e)  Agriculteur exploitant  Artisan  Commerçant et assimilé  Cadre d'entreprise, profession intermédiaire de la santé, fonction publique et assimilé  Profession intermédiaire administrative et commerciale des entreprises  Technicien |
|  | Occ_stat_3=3 | Attaché  Conseiller d'éducation  Secrétaire  Commis, agent ou adjoint administratif  Sténo-dactylo  Ouvrier contremaître  Agent de bureau  Agent d'établissement d'enseignement  Autre : en clair NOENSAUT_CAR  Contremaître - Agent de maîtrise  Employé de la fonction publique  Employé administratif d'entreprise  Employé de commerce  Personnel des services directs aux particuliers  Ouvrier qualifié  Ouvrier non qualifié  Ouvrier agricole  CNAS |
| **EPIC Italy** | Occ_stat_3=1 | professionals and managers |
|  | Occ_stat_3=2 | farmer  retailer  clerical worker |
|  | Occ_stat_3=3 | unskilled workers  skilled workers |
| **EPIPORTO** | Occ_stat_3=1 | Higher Professions/occupations |
|  | Occ_stat_3=2 | Intermediate Professions/occupations  Skilled agricultural and fishery workers |
|  | Occ_stat_3=3 | Non-manual skilled occupations  Semi-skilled occupations  Unskilled occupations |
| **GAZEL** | Occ_stat_3=1 | Administrative executive  Technical executive |
|  | Occ_stat_3=2 | Administrative supervisor  Technical supervisor  Teacher, school staff |
|  | Occ_stat_3=3 | Sales representative  Patrolman, block manager  Labourer  Employee  Trainee agent |
| **SKIPOGH** | Occ_stat_3=1 | Self-employed, liberal professions (doctors, lawyers,…)  Higher level managers (directors, managers,…)  Executive managers (engineer, magistrate,…)  Professors, headmasters  Clergy |
|  | Occ_stat_3=2 | Self-employed, commerce and trade (butcher, shoemaker, hairdresser,…)  Skilled workers in the health service (nurse, physiotherapist, laboratory worker,…)  Farmer  Teachers, educators  Public safety, public force (military, policemen, firemen) |
|  | Occ_stat_3=3 | Skilled non-manual worker (secretary, technician, programmer,…)  Unskilled non-manual worker (waiter, postman, clerk,…)  Skilled manual worker (metalworker, mechanic, plumber, miner,…)  Unskilled manual worker (carrier, driver, cleaner) |
| **TILDA** | Occ_stat_3=1 | Professional workers  Managerial & technical |
|  | Occ_stat_3=2 | Farmers |
|  | Occ_stat_3=3 | Non-manual  Skilled manual  Semi-skilled  Unskilled |
| **WHIP-retired** | Occ_stat_3=1 | Supervisor  Manager |
|  | Occ_stat_3=2 | Employee |
|  | Occ_stat_3=3 | Apprentice  Laborer |
| **Whitehall II** | Occ_stat_3=1 | Unified Grade 1–6 at right wave  Unified Grade 7 at right wave  Senior Executive Officer at right wave |
|  | Occ_stat_3=2 | Higher Executive Officer at right wave  Executive Officer at right wave |
|  | Occ_stat_3=3 | Clerical and Support Staff, e.g. messengers, porters, telephonists, typists at right wave |
