# Supplementary material for: Socioeconomic indicators in epidemiologic research: A practical example from the LIFEPATH study
Source: PLoS One. 2017 May 30;12(5):e0178071. doi: 10.1371/journal.pone.0178071 (PMC5448763; doi:10.1371/journal.pone.0178071)
Supplement: S4 File — (DOC) [file pone.0178071.s004.doc]

**S4 File. Harmonization of FATHER’S JOB ***

***only GAZEL, SKIPOGH and WHITEHALL cohorts codified subjects’ job and fathers’ job using different codes**

**Two levels variable**

occ_f_2 =1 🡪 manual workers

occ_f_2 =2 🡪 non manual workers

| **Constances** | Occ_f_2=1 | Farmer  Worker |
| --- | --- | --- |
|  | Occ_f_2=2 | Employer  Manager, intellectual profession  Middle profession  Clerical or service employee |
| **E3N** | Occ_f_2=1 | Ouvrier contremaître  Agriculteur exploitant  Artisan  Contremaître - Agent de maîtrise  Personnel des services directs aux particuliers  Ouvrier qualifié  Ouvrier non qualifié  Ouvrier agricole  CNAS |
|  | Occ_f_2=2 | Directeur d'école 1er degré  Instituteur  Agrégé  Certifié  Adjoint d'enseignement  PEGC  Professeur de lycée professionnel  Maître auxiliaire  Professeur d'Université  Maître de conférence des Universités  Direction - Inspection  Chef d'établissement 2nd degré  Attaché  Conseiller d'éducation  Secrétaire  Infirmier( e)  Commis, agent ou adjoint administratif  Sténo-dactylo  Agent de bureau  Agent d'établissement d'enseignement  Autre : en clair NOENSAUT_CAR  Commerçant et assimilé  Chef d'entreprise (10 salariés ou +)  Profession libérale  Cadre fonction publique, profession intellectuelle, artistique  Cadre d'entreprise, profession intermédiaire de la santé, fonction publique et assimilé  Profession intermédiaire administrative et commerciale des entreprises  Technicien  Employé de la fonction publique  Employé administratif d'entreprise  Employé de commerce |
| **EPIC Italy** | Occ_f_2=1 | farmer  unskilled workers  skilled workers |
|  | Occ_f_2=2 | retailer  clerical worker  professionals and managers |
| **EPIPORTO** | Occ_f_2=1 | Skilled agricultural and fishery workers  Semi-skilled occupations  Unskilled occupations |
|  | Occ_f_2=2 | Higher Professions/occupations  Intermediate Professions/occupations  Non-manual skilled occupations |
| **GAZEL** | Occ_f_2=1 | farmer  Employee  Labourer |
|  | Occ_f_2=2 | Craftsman, shopkeeper  Business owner (10 or more employees)  Executive  Intermediary |
| **SKIPOGH** | Occ_f_2=1 | Labourer, worker  Qualified worker, foreman (supervisor)  Farmer  Employee without qualification (ex. Unqualified office clerk) |
|  | Occ_f_2=2 | Qualified employee (ex. Secretary, accountant)  Middle manager (ex. Technician, Teacher)  Small business self-employed, craftsman  Executive manager (ex. Economist, legal expert in a company)  Liberal profession (ex. Physician, legal expert (self-employed)) |
| **TILDA** | Occ_f_2=1 | Skilled manual  Semi-skilled  Unskilled  Farmers |
|  | Occ_f_2=2 | Professional workers  Managerial & technical  Non-manual |
| **Whitehall II** | Occ_f_2=1 | Skilled occupations, manual (IIIM)  Semi-skilled (IV)  Unskilled (V) |
|  | Occ_f_2=2 | Professional occupations (I)  Managerial & technical occupations (II)  Skilled occupations, non-manual (IIIN) |

**Three levels variable**

occ_f_3 =1 🡪 higher professionals and managers, lower professionals and managers; higher clerical, services and sales workers (Class 1-3 ESEC – European Socio-economic Classification – 9 classes)

occ_f_3=2 🡪 small employers and self-employed; farmers; lower supervisors and technicians (Class 4, 5, and 6 ESEC) –include here intermediate professions such as teachers, nurses, etc

occ_f_3=3 🡪 lower clerical, services, and sales workers; skilled workers; semi – and unskilled workers (Class 7-9 ESEC)

| **Constances** | Occ_f_3=1 | Manager, intellectual profession |
| --- | --- | --- |
|  | Occ_f_3=2 | Farmer  Employer  Middle profession |
|  | Occ_f_3=3 | Clerical or service employee  Worker |
| **E3N** | Occ_f_3=1 | Directeur d'école 1er degré  Maître auxiliaire  Professeur d'Université  Maître de conférence des Universités  Direction - Inspection  Chef d'établissement 2nd degré  Chef d'entreprise (10 salariés ou +)  Profession libérale  Cadre fonction publique, profession intellectuelle, artistique |
|  | Occ_f_3=2 | Instituteur  Agrégé  Certifié  Adjoint d'enseignement  PEGC  Professeur de lycée professionnel  Infirmier( e)  Agriculteur exploitant  Artisan  Commerçant et assimilé  Cadre d'entreprise, profession intermédiaire de la santé, fonction publique et assimilé  Profession intermédiaire administrative et commerciale des entreprises  Technicien |
|  | Occ_f_3=3 | Attaché  Conseiller d'éducation  Secrétaire  Commis, agent ou adjoint administratif  Sténo-dactylo  Ouvrier contremaître  Agent de bureau  Agent d'établissement d'enseignement  Autre : en clair NOENSAUT_CAR  Contremaître - Agent de maîtrise  Employé de la fonction publique  Employé administratif d'entreprise  Employé de commerce  Personnel des services directs aux particuliers  Ouvrier qualifié  Ouvrier non qualifié  Ouvrier agricole  CNAS |
| **EPIC Italy** | Occ_f_3=1 | professionals and managers |
|  | Occ_f_3=2 | farmer  retailer  clerical worker |
|  | Occ_f_3=3 | unskilled workers  skilled workers |
| **GAZEL** | Occ_f_3=1 | Business owner (10 or more employees)  Executive  Intermediary |
|  | Occ_f_3=2 | farmer  Craftsman, shopkeeper |
|  | Occ_f_3=3 | Employee  Labourer |
| **SKIPOGH** | Occ_f_3=1 | Executive manager (ex. Economist, legal expert in a company)  Liberal profession (ex. Physician, legal expert (self-employed))  Director, CEO of a company or of a public service |
|  | Occ_f_3=2 | Farmer  Qualified employee (ex. Secretary, accountant)  Middle manager (ex. Technician, Teacher)  Small business self-employed, craftsman |
|  | Occ_f_3=3 | Labourer, worker  Qualified worker, foreman (supervisor)  Employee without qualification (ex. Unqualified office clerk) |
| **TILDA** | Occ_f_3=1 | Professional workers  Managerial & technical |
|  | Occ_f_3=2 | Farmers |
|  | Occ_f_3=3 | Non-manual  Skilled manual  Semi-skilled  Unskilled |
| **Whitehall II** | Occ_f_3=1 | Professional occupations (I) |
|  | Occ_f_3=2 | Managerial & technical occupations (II) |
|  | Occ_f_3=3 | Skilled occupations, non-manual (IIIN)  Skilled occupations, manual (IIIM)  Semi-skilled (IV) |
