# Supplementary material for: Socioeconomic indicators in epidemiologic research: A practical example from the LIFEPATH study
Source: PLoS One. 2017 May 30;12(5):e0178071. doi: 10.1371/journal.pone.0178071 (PMC5448763; doi:10.1371/journal.pone.0178071)
Supplement: S1 Table — Males. (DOC) [file pone.0178071.s005.doc]

**S1 Table. Association between socioeconomic variables and mortality separated by cohort. Males**

|  | **CoLaus** | | | **EPIC-Italy** | | | **EPIPORTO** | | | **Gazel** | | | **MCCS** | | | **WHIP retired** | | | **WHITEHALL II** | | |
| --- | --- | --- | --- | --- | --- | --- | --- | --- | --- | --- | --- | --- | --- | --- | --- | --- | --- | --- | --- | --- | --- |
|  | **RR** | **Low** | **Up** | **RR** | **Low** | **Up** | **RR** | **Low** | **Up** | **RR** | **Low** | **Up** | **RR** | **Low** | **Up** | **RR** | **Low** | **Up** | **RR** | **Low** | **Up** |
| **Edulev 3** | | | | | | | | | | | | | | | | | | | | | |
| *tertiary* | Ref | | | Ref | | | Ref | | | Ref | | | Ref | | |  |  |  | Ref | | |
| *secondary* | 0.98 | 0.55 | 1.74 | 1.29 | 0.99 | 1.68 | 2.02 | 1.08 | 3.76 | 1.42 | 1.18 | 1.72 | 1.26 | 1.15 | 1.38 |  |  |  | 1.03 | 0.87 | 1.22 |
| *primary* | 1.32 | 0.88 | 1.99 | 1.59 | 1.26 | 2.02 | 2.05 | 1.18 | 3.55 | 1.57 | 1.38 | 1.79 | 1.29 | 1.21 | 1.38 |  |  |  | 1.07 | 0.92 | 1.26 |
| **Edulev 4** | | | | | | | | | | | | | | | | | | | | | |
| *tertiary* | Ref | | | Ref | | |  |  |  | Ref | | |  |  |  |  |  |  |  |  |  |
| *secondary* | 0.98 | 0.55 | 1.74 | 1.29 | 0.99 | 1.68 |  |  |  | 1.42 | 1.17 | 1.72 |  |  |  |  |  |  |  |  |  |
| *vocational* | 1.28 | 0.83 | 1.96 | 1.42 | 1.07 | 1.87 |  |  |  | 1.51 | 1.32 | 1.72 |  |  |  |  |  |  |  |  |  |
| *primary* | 1.44 | 0.87 | 2.40 | 1.66 | 1.30 | 2.11 |  |  |  | 1.75 | 1.51 | 2.03 |  |  |  |  |  |  |  |  |  |
| **Employment status** | | | | | | | | | | | | | | | | | | | | | |
| *employed* | Ref | | | Ref | | | Ref | | | Ref | | | Ref | | |  |  |  | Ref | | |
| *not employed* | 2.31 | 1.44 | 3.71 | 1.22 | 1.03 | 1.44 | 1.61 | 1.05 | 2.49 | 1.00 | 1.00 | 1.00 | 1.51 | 1.38 | 1.66 |  |  |  | 1.56 | 1.26 | 1.93 |
| **Employment status** | | | | | | | | | | | | | | | | | | | | | |
| *employed* |  |  |  |  |  |  |  |  |  |  |  |  | Ref | | |  |  |  |  |  |  |
| *Not employed: retired* |  |  |  |  |  |  |  |  |  |  |  |  | 1.53 | 1.39 | 1.68 |  |  |  |  |  |  |
| *Not employed: housewife* |  |  |  |  |  |  |  |  |  |  |  |  | 1.19 | 0.77 | 1.83 |  |  |  |  |  |  |
| *Not employed: unemployed* |  |  |  |  |  |  |  |  |  |  |  |  | 1.53 | 1.27 | 1.85 |  |  |  |  |  |  |
| *Not employed: disabled* |  |  |  |  |  |  |  |  |  |  |  |  | NA | NA | NA |  |  |  |  |  |  |
| **Occ stat 2** | | | | | | | | | | | | | | | | | | | | | |
| *non manual* | Ref | | | Ref | | | Ref | | | Ref | | |  |  |  |  |  |  | Ref | | |
| *Manual* | 1.44 | 0.77 | 2.69 | 1.18 | 0.98 | 1.42 | 0.90 | 0.46 | 1.76 | 1.70 | 1.53 | 1.90 |  |  |  |  |  |  | 1.55 | 1.26 | 1.91 |
| **Occ stat 3** | | | | | | | | | | | | | | | | | | | | | |
| *Classes 1-3 ESEC* | Ref | | | Ref | | | Ref | | | Ref | | |  |  |  |  |  |  | Ref | | |
| *Classes 4-6 ESEC* | 1.41 | 0.60 | 3.27 | 1.38 | 0.98 | 1.95 | 1.07 | 0.38 | 2.97 | 1.45 | 1.29 | 1.62 |  |  |  |  |  |  | 1.26 | 1.08 | 1.46 |
| *Classes 7-9 ESEC* | 2.13 | 0.98 | 4.61 | 1.55 | 1.09 | 2.20 | 1.79 | 0.70 | 4.58 | 1.99 | 1.76 | 2.26 |  |  |  |  |  |  | 1.67 | 1.34 | 2.07 |
| **Occ last 2** | | | | | | | | | | | | | | | | | | | | | |
| *non manual* | Ref | | | Ref | | | Ref | | | Ref | | |  |  |  | Ref | | | Ref | | |
| *Manual* | 1.38 | 0.74 | 2.58 | 1.19 | 1.05 | 1.35 | 1.07 | 0.81 | 1.41 | 1.70 | 1.53 | 1.90 |  |  |  | 1.28 | 1.24 | 1.31 | 1.66 | 1.37 | 2.00 |
| **Occ last 3** | | | | | | | | | | | | | | | | | | | | | |
| *Classes 1-3 ESEC* | Ref | | | Ref | | | Ref | | | Ref | | |  |  |  | Ref | | | Ref | | |
| *Classes 4-6 ESEC* | 1.38 | 0.61 | 3.14 | 1.19 | 0.92 | 1.54 | 1.43 | 0.87 | 2.34 | 1.45 | 1.29 | 1.62 |  |  |  | 1.60 | 1.47 | 1.74 | 1.28 | 1.11 | 1.48 |
| *Classes 7-9 ESEC* | 2.14 | 1.01 | 4.51 | 1.37 | 1.05 | 1.78 | 1.64 | 1.03 | 2.62 | 1.99 | 1.76 | 2.26 |  |  |  | 1.93 | 1.78 | 2.10 | 1.79 | 1.47 | 2.17 |
| **Occ f 2** | | | | | | | | | | | | | | | | | | | | | |
| *non manual* |  |  |  | Ref | | | Ref | | | Ref | | |  |  |  |  |  |  | Ref | | |
| *Manual* |  |  |  | 1.00 | 0.87 | 1.16 | 1.06 | 0.69 | 1.60 | 0.97 | 0.88 | 1.06 |  |  |  |  |  |  | 1.14 | 0.97 | 1.32 |
| **Occ f 3** | | | | | | | | | | | | | | | | | | | | | |
| *Classes 1-3 ESEC* |  |  |  | Ref | | | Ref | | | Ref | | |  |  |  |  |  |  | Ref | | |
| *Classes 4-6 ESEC* |  |  |  | 1.18 | 0.85 | 1.64 | 1.89 | 0.81 | 4.39 | 0.89 | 0.78 | 1.01 |  |  |  |  |  |  | 0.86 | 0.64 | 1.16 |
| *Classes 7-9 ESEC* |  |  |  | 1.22 | 0.87 | 1.70 | 2.36 | 1.10 | 5.04 | 1.01 | 0.91 | 1.12 |  |  |  |  |  |  | 1.08 | 0.82 | 1.42 |
| **Income 5** | | | | | | | | | | | | | | | | | | | | | |
| *Fifth quintile* | Ref | | |  |  |  |  |  |  |  |  |  |  |  |  | Ref | | |  |  |  |
| *Fourth quintile* | 2.17 | 0.67 | 7.08 |  |  |  |  |  |  |  |  |  |  |  |  | 1.16 | 1.12 | 1.20 |  |  |  |
| *Third quintale* | 0.96 | 0.23 | 3.96 |  |  |  |  |  |  |  |  |  |  |  |  | 1.26 | 1.22 | 1.31 |  |  |  |
| *Second quintile* | 3.17 | 1.11 | 9.06 |  |  |  |  |  |  |  |  |  |  |  |  | 1.28 | 1.23 | 1.32 |  |  |  |
| *First quintile* | 3.86 | 1.36 | 10.98 |  |  |  |  |  |  |  |  |  |  |  |  | 1.25 | 1.19 | 1.32 |  |  |  |
