# Supplementary material for: Socioeconomic indicators in epidemiologic research: A practical example from the LIFEPATH study
Source: PLoS One. 2017 May 30;12(5):e0178071. doi: 10.1371/journal.pone.0178071 (PMC5448763; doi:10.1371/journal.pone.0178071)
Supplement: S2 Table — Females. (DOC) [file pone.0178071.s006.doc]

**S2 Table. Association between socioeconomic variables and mortality separated by cohort. Females**

|  | **CoLaus** | | | **E3N** | | | **EPIC-Italy** | | | **EPIPORTO** | | | **Gazel** | | | **MCCS** | | | **WHIP retired** | | | **WHITEHALL II** | | |
| --- | --- | --- | --- | --- | --- | --- | --- | --- | --- | --- | --- | --- | --- | --- | --- | --- | --- | --- | --- | --- | --- | --- | --- | --- |
|  | **IRR** | **Low** | **Up** | **IRR** | **Low** | **Up** | **IRR** | **Low** | **Up** | **IRR** | **Low** | **Up** | **IRR** | **Low** | **Up** | **IRR** | **Low** | **Up** | **IRR** | **Low** | **Up** | **IRR** | **Low** | **Up** |
| **Edulev 3** | | | | | | | | | | | | | | | | | | | | | | | | |
| *tertiary* | Ref | | | Ref | | | Ref | | | Ref | | | Ref | | | Ref | | |  | | | Ref | | |
| *secondary* | 1.47 | 0.60 | 3.57 | 1.00 | 0.96 | 1.04 | 1.03 | 0.81 | 1.32 | 0.96 | 0.33 | 2.75 | 1.17 | 0.76 | 1.83 | 1.08 | 0.96 | 1.21 |  |  |  | 1.25 | 0.90 | 1.74 |
| *primary* | 1.57 | 0.81 | 3.03 | 1.26 | 1.17 | 1.36 | 1.03 | 0.83 | 1.27 | 1.59 | 0.75 | 3.37 | 1.17 | 0.83 | 1.67 | 1.10 | 1.01 | 1.19 |  |  |  | 1.03 | 0.77 | 1.36 |
| **Edulev 4** | | | | | | | | | | | | | | | | | | | | | | | | |
| *tertiary* | Ref | | | Ref | | | Ref | | |  | | | Ref | | |  | | |  | | |  | | |
| *secondary* | 1.47 | 0.61 | 3.58 | 0.99 | 0.94 | 1.03 | 1.03 | 0.81 | 1.32 |  |  |  | 1.17 | 0.76 | 1.83 |  |  |  |  |  |  |  |  |  |
| *vocational* | 1.23 | 0.60 | 2.51 | 1.07 | 0.99 | 1.15 | 1.00 | 0.75 | 1.33 |  |  |  | 1.17 | 0.82 | 1.67 |  |  |  |  |  |  |  |  |  |
| *primary* | 2.06 | 1.02 | 4.18 | 1.26 | 1.17 | 1.36 | 1.04 | 0.84 | 1.28 |  |  |  | 1.19 | 0.81 | 1.74 |  |  |  |  |  |  |  |  |  |
| **Employment status** | | | | | | | | | | | | | | | | | | | | | | | | |
| *employed* | Ref | | | Ref | | | Ref | | | Ref | | |  | | | Ref | | | Ref | | | Ref | | |
| *not employed* | 1.91 | 1.06 | 3.45 | 1.14 | 1.08 | 1.20 | 0.98 | 0.83 | 1.16 | 2.59 | 1.19 | 5.60 |  |  |  | 1.18 | 1.05 | 1.32 | 1.00 | 1.00 | 1.00 | 1.64 | 1.25 | 2.15 |
| **Employment status** | | | | | | | | | | | | | | | | | | | | | | | | |
| *employed* |  | | |  | | |  | | |  | | |  | | | Ref | | |  | | |  | | |
| *Not employed: retired* |  |  |  |  |  |  |  |  |  |  |  |  |  |  |  | 1.19 | 1.06 | 1.34 |  |  |  |  |  |  |
| *Not employed: housewife* |  |  |  |  |  |  |  |  |  |  |  |  |  |  |  | 1.11 | 0.99 | 1.25 |  |  |  |  |  |  |
| *Not employed: unemployed* |  |  |  |  |  |  |  |  |  |  |  |  |  |  |  | 1.63 | 1.20 | 2.21 |  |  |  |  |  |  |
| *Not employed: disabled* |  |  |  |  |  |  |  |  |  |  |  |  |  |  |  | NA | NA | NA |  |  |  |  |  |  |
| **Occ stat 2** | | | | | | | | | | | | | | | | | | | | | | | | |
| *non manual* | Ref | | | Ref | | | Ref | | | Ref | | | Ref | | |  | | | Ref | | | Ref | | |
| *Manual* | 1.52 | 0.57 | 4.04 | 0.98 | 0.78 | 1.23 | 1.18 | 0.90 | 1.53 | 0.78 | 0.17 | 3.65 | 1.03 | 0.82 | 1.30 |  |  |  | 1.00 | 1.00 | 1.00 | 1.08 | 0.88 | 1.31 |
| **Occ stat 3** | | | | | | | | | | | | | | | | | | | | | | | | |
| *Classes 1-3 ESEC* | Ref | | | Ref | | | Ref | | | Ref | | | Ref | | |  | | | Ref | | | Ref | | |
| *Classes 4-6 ESEC* | 1.56 | 0.19 | 13.03 | 1.06 | 1.00 | 1.13 | 0.92 | 0.51 | 1.64 | 0.76 | 0.07 | 8.09 | 1.00 | 0.68 | 1.46 |  |  |  | 1.00 | 1.00 | 1.00 | 1.15 | 0.85 | 1.56 |
| *Classes 7-9 ESEC* | 1.93 | 0.26 | 14.54 | 1.15 | 1.06 | 1.24 | 1.02 | 0.56 | 1.86 | 0.84 | 0.16 | 4.38 | 1.01 | 0.68 | 1.51 |  |  |  | 1.00 | 1.00 | 1.00 | 1.19 | 0.88 | 1.61 |
| **Occ last 2** | | | | | | | | | | | | | | | | | | | | | | | | |
| *non manual* | Ref | | |  | | | Ref | | | Ref | | | Ref | | |  | | | Ref | | | Ref | | |
| *Manual* | 0.95 | 0.41 | 2.23 |  |  |  | 1.13 | 0.96 | 1.33 | 2.05 | 1.28 | 3.29 | 1.03 | 0.82 | 1.30 |  |  |  | 1.03 | 0.95 | 1.12 | 1.08 | 0.89 | 1.30 |
| **Occ last 3** | | | | | | | | | | | | | | | | | | | | | | | | |
| *Classes 1-3 ESEC* | Ref | | |  | | | Ref | | | Ref | | | Ref | | |  | | | Ref | | | Ref | | |
| *Classes 4-6 ESEC* | 2.19 | 0.28 | 17.32 |  |  |  | 0.96 | 0.60 | 1.54 | 1.09 | 0.23 | 5.27 | 1.00 | 0.68 | 1.46 |  |  |  | 1.30 | 0.92 | 1.85 | 1.17 | 0.87 | 1.56 |
| *Classes 7-9 ESEC* | 2.14 | 0.29 | 15.97 |  |  |  | 1.07 | 0.67 | 1.70 | 2.80 | 0.70 | 11.22 | 1.01 | 0.68 | 1.51 |  |  |  | 1.33 | 0.94 | 1.88 | 1.20 | 0.90 | 1.60 |
| **Occ f 2** | | | | | | | | | | | | | | | | | | | | | | | | |
| *non manual* |  | | | Ref | | | Ref | | | Ref | | | Ref | | |  | | |  | | | Ref | | |
| *Manual* |  |  |  | 1.01 | 0.96 | 1.06 | 1.00 | 0.86 | 1.15 | 0.86 | 0.42 | 1.75 | 0.96 | 0.78 | 1.19 |  |  |  |  |  |  | 1.02 | 0.81 | 1.29 |
| **Occ f 3** | | | | | | | | | | | | | | | | | | | | | | | | |
| *Classes 1-3 ESEC* |  | | | Ref | | | Ref | | | Ref | | | Ref | | |  | | |  | | | Ref | | |
| *Classes 4-6 ESEC* |  |  |  | 0.96 | 0.90 | 1.03 | 1.05 | 0.77 | 1.43 | 2.83 | 0.34 | 23.17 | 0.75 | 0.55 | 1.02 |  |  |  |  |  |  | 0.97 | 0.62 | 1.53 |
| *Classes 7-9 ESEC* |  |  |  | 1.04 | 0.97 | 1.12 | 1.03 | 0.76 | 1.41 | 3.15 | 0.43 | 23.35 | 1.08 | 0.86 | 1.36 |  |  |  |  |  |  | 0.87 | 0.57 | 1.33 |
| **Income 5** | | | | | | | | | | | | | | | | | | | | | | | | |
| *Fifth quintile* |  | | |  | | |  | | |  | | |  | | |  | | |  | | |  | | |
| *Fourth quintile* |  |  |  |  |  |  |  |  |  |  |  |  |  |  |  |  |  |  | 1.01 | 0.87 | 1.16 |  |  |  |
| *Third quintale* |  |  |  |  |  |  |  |  |  |  |  |  |  |  |  |  |  |  | 1.07 | 0.94 | 1.22 |  |  |  |
| *Second quintile* |  |  |  |  |  |  |  |  |  |  |  |  |  |  |  |  |  |  | 1.14 | 1.02 | 1.28 |  |  |  |
| *First quintile* |  |  |  |  |  |  |  |  |  |  |  |  |  |  |  |  |  |  | 1.06 | 0.94 | 1.20 |  |  |  |
