# Supplementary material for: Socioeconomic indicators in epidemiologic research: A practical example from the LIFEPATH study
Source: PLoS One. 2017 May 30;12(5):e0178071. doi: 10.1371/journal.pone.0178071 (PMC5448763; doi:10.1371/journal.pone.0178071)
Supplement: S1 Fig — (DOC) [file pone.0178071.s007.doc]

**S1 Fig. Meta-analysis of the association between current/last job and mortality separating skilled and semi- and unskilled workers.**

**PANEL A – Males**

NOTE: Weights are from random effects analysis

Overall (I-squared = 46.2%, p = 0.134)

Gazel

EPIC Italy

EPIPORTO

CoLaus

ID

Study

1.65 (1.29, 2.02)

1.85 (1.60, 2.15)

1.33 (1.02, 1.74)

1.71 (1.05, 2.81)

2.37 (1.13, 4.96)

RR (95% CI)

100.00

45.06

38.22

13.30

3.41

Weight

%

1.65 (1.29, 2.02)

1.85 (1.60, 2.15)

1.33 (1.02, 1.74)

1.71 (1.05, 2.81)

2.37 (1.13, 4.96)

100.00

45.06

38.22

13.30

3.41

Weight

%

1

-4.96

1

4.96

**Classes 7-8 ESEC vs Classes 1-3 ESEC**

NOTE: Weights are from random effects analysis

Overall (I-squared = 55.1%, p = 0.083)

Gazel

ID

EPIPORTO

EPIC Italy

CoLaus

Study

1.76 (1.29, 2.23)

2.16 (1.86, 2.52)

RR (95% CI)

1.57 (0.95, 2.61)

1.49 (1.09, 2.03)

1.11 (0.30, 4.04)

100.00

40.86

Weight

19.63

33.90

5.60

%

1.76 (1.29, 2.23)

2.16 (1.86, 2.52)

1.57 (0.95, 2.61)

1.49 (1.09, 2.03)

1.11 (0.30, 4.04)

100.00

40.86

Weight

19.63

33.90

5.60

%

1

-4.04

1

4.04

**Classes 9 ESEC vs Classes 1-3 ESEC**

**PANEL B – Females**

NOTE: Weights are from random effects analysis

Overall (I-squared = 0.0%, p = 0.964)

Gazel

Study

CoLaus

ID

EPIPORTO

EPIC Italy

1.00 (0.69, 1.32)

1.01 (0.68, 1.51)

1.78 (0.23, 13.54)

RR (95% CI)

1.96 (0.44, 8.69)

0.97 (0.60, 1.56)

100.00

56.58

%

0.22

Weight

0.57

42.63

1.00 (0.69, 1.32)

1.01 (0.68, 1.51)

1.78 (0.23, 13.54)

1.96 (0.44, 8.69)

0.97 (0.60, 1.56)

100.00

56.58

%

0.22

Weight

0.57

42.63

1

-13.5

1

13.5

**Classes 7-8 ESEC vs Classes 1-3 ESEC**

NOTE: Weights are from random effects analysis

Overall (I-squared = 0.0%, p = 0.873)

ID

Gazel

EPIC Italy

EPIPORTO

CoLaus

Study

1.22 (0.63, 1.80)

RR (95% CI)

0.88 (0.21, 3.76)

1.23 (0.76, 2.00)

3.19 (0.80, 12.73)

8.55 (0.94, 77.88)

100.00

Weight

10.79

88.24

0.96

0.02

%

1.22 (0.63, 1.80)

0.88 (0.21, 3.76)

1.23 (0.76, 2.00)

3.19 (0.80, 12.73)

8.55 (0.94, 77.88)

100.00

Weight

10.79

88.24

0.96

0.02

%

1

-77.9

1

77.9

**Classes 9 ESEC vs Classes 1-3 ESEC**
