# Supplementary material for: Socioeconomic indicators in epidemiologic research: A practical example from the LIFEPATH study
Source: PLoS One. 2017 May 30;12(5):e0178071. doi: 10.1371/journal.pone.0178071 (PMC5448763; doi:10.1371/journal.pone.0178071)
Supplement: S2 Fig — (DOC) [file pone.0178071.s008.doc]

**S2 Fig. Meta-analysis of the association between fathers’ job and mortality separating skilled and semi- and unskilled workers.**

**PANEL A – Males**

NOTE: Weights are from random effects analysis

Overall (I-squared = 31.2%, p = 0.225)

EPIPORTO

ID

Whitehall II

Gazel

Study

EPIC Italy

1.01 (0.80, 1.21)

2.21 (0.96, 5.06)

RR (95% CI)

1.07 (0.81, 1.41)

0.87 (0.72, 1.06)

1.21 (0.86, 1.70)

100.00

0.99

Weight

29.77

50.75

%

18.48

1.01 (0.80, 1.21)

2.21 (0.96, 5.06)

1.07 (0.81, 1.41)

0.87 (0.72, 1.06)

1.21 (0.86, 1.70)

100.00

0.99

Weight

29.77

50.75

%

18.48

1

-5.06

1

5.06

**Classes 7-8 ESEC vs Classes 1-3 ESEC**

NOTE: Weights are from random effects analysis

Overall (I-squared = 0.0%, p = 0.410)

EPIC Italy

Whitehall II

Gazel

ID

EPIPORTO

Study

1.06 (0.96, 1.17)

1.23 (0.86, 1.75)

1.27 (0.84, 1.94)

1.04 (0.94, 1.16)

RR (95% CI)

2.45 (1.12, 5.36)

100.00

5.54

3.58

90.64

Weight

0.24

%

1.06 (0.96, 1.17)

1.23 (0.86, 1.75)

1.27 (0.84, 1.94)

1.04 (0.94, 1.16)

2.45 (1.12, 5.36)

100.00

5.54

3.58

90.64

Weight

0.24

%

1

-5.36

1

5.36

**Classes 9 ESEC vs Classes 1-3 ESEC**

**PANEL B – Females**

NOTE: Weights are from random effects analysis

Overall (I-squared = 0.0%, p = 0.983)

ID

Whitehall II

EPIPORTO

Gazel

EPIC Italy

Study

1.04 (0.97, 1.11)

RR (95% CI)

1.01 (0.68, 1.50)

1.04 (0.75, 1.42)

3.92 (0.51, 29.95)

1.04 (0.97, 1.12)

100.00

Weight

3.00

4.52

0.00

92.47

%

1.04 (0.97, 1.11)

1.01 (0.68, 1.50)

1.04 (0.75, 1.42)

3.92 (0.51, 29.95)

1.04 (0.97, 1.12)

100.00

Weight

3.00

4.52

0.00

92.47

%

1

-30

1

30

**Classes 7-8 ESEC vs Classes 1-3 ESEC**

NOTE: Weights are from random effects analysis

Overall (I-squared = 0.0%, p = 0.963)

Whitehall II

EPIC Italy

Gazel

Study

EPIPORTO

ID

1.05 (0.94, 1.16)

1.10 (0.86, 1.40)

1.04 (0.92, 1.17)

2.60 (0.33, 20.35)

1.02 (0.72, 1.44)

RR (95% CI)

100.00

16.51

74.39

0.01

%

9.09

Weight

1.05 (0.94, 1.16)

1.10 (0.86, 1.40)

1.04 (0.92, 1.17)

2.60 (0.33, 20.35)

1.02 (0.72, 1.44)

100.00

16.51

74.39

0.01

%

9.09

Weight

1

-20.4

1

20.4

**Classes 9 ESEC vs Classes 1-3 ESEC**
